# MoCAP proteins regulated by MoArk1-mediated phosphorylation coordinate endocytosis and actin dynamics to govern development and virulence of *Magnaporthe oryzae*

**DOI:** 10.1371/journal.pgen.1006814

**Published:** 2017-05-25

**Authors:** Lianwei Li, Xiaolin Chen, Shengpei Zhang, Jun Yang, Deng Chen, Muxing Liu, Haifeng Zhang, Xiaobo Zheng, Ping Wang, Youliang Peng, Zhengguang Zhang

**Affiliations:** 1 Department of Plant Pathology, College of Plant Protection, Nanjing Agricultural University, and Key Laboratory of Integrated Management of Crop Diseases and Pests, Ministry of Education, Nanjing, China; 2 State Key Laboratory of Agrobiotechnology and Ministry of Agriculture Key Laboratory of Plant Pathology, China Agricultural University, Beijing, China; 3 Departments of Pediatrics, and Microbiology, Immunology, and Parasitology, Louisiana State University Health Sciences Center, New Orleans, Louisiana, United States of America; Karlsruhe Institute of Technology, GERMANY

## Abstract

Actin organization is a conserved cellular process that regulates the growth and development of eukaryotic cells. It also governs the virulence process of pathogenic fungi, such as the rice blast fungus *Magnaporthe oryzae*, with mechanisms not yet fully understood. In a previous study, we found that actin-regulating kinase MoArk1 displays conserved functions important in endocytosis and actin organization, and MoArk1 is required for maintaining the growth and full virulence of *M*. *oryzae*. To understand how MoArk1 might function, we identified capping protein homologs from *M*. *oryzae* (MoCAP) that interact with MoArk1 *in vivo*. MoCAP is heterodimer consisting of α and β subunits MoCapA and MoCapB. Single and double deletions of MoCAP subunits resulted in abnormal mycelial growth and conidia formation. The Δ*Mocap* mutants also exhibited reduced appressorium penetration and invasive hyphal growth within host cells. Furthermore, the Δ*Mocap* mutants exhibited delayed endocytosis and abnormal cytoskeleton assembly. Consistent with above findings, MoCAP proteins interacted with MoAct1, co-localized with actin during mycelial development, and participated in appressorial actin ring formation. Further analysis revealed that the S85 residue of MoCapA and the S285 residue of MoCapB were subject to phosphorylation by MoArk1 that negatively regulates MoCAP functions. Finally, the addition of exogenous phosphatidylinositol 4,5-bisphosphate (PIP_2_) failed to modulate actin ring formation in Δ*Mocap* mutants, in contrast to the wild-type strain, suggesting that MoCAP may also mediate phospholipid signaling in the regulation of the actin organization. These results together demonstrate that MoCAP proteins whose functions are regulated by MoArk1 and PIP_2_ are important for endocytosis and actin dynamics that are directly linked to growth, conidiation and pathogenicity of *M*. *oryzae*.

## Author summary

The actin-regulating kinase MoArk1 plays a conserved function in endocytosis and actin organization and is also essential for growth and full virulence of the rice blast fungus *Magnaporthe oryzae*. To understand how MoArk1 functions, we identified the F-actin capping protein α (MoCapA) and β (MoCapB) subunits that interact with MoArk1. We showed that single and double deletions of *MoCAPA* and *MoCAPB* result in slowed growth, reduced conidia production, abnormal morphogenesis, and attenuated virulence. We found that Δ*Mocap* mutants are defective in endocytosis and actin organization and that MoCAP proteins are subject to regulation by MoArk1 through protein phosphorylation. Finally, we provided evidence demonstrating that MoCAP proteins modulate actin dynamics in response to phosphatidylinositol 4,5-biphosphate (PIP_2_). These combined results suggest that MoCAP proteins play an important role in endocytosis, actin organization, and virulence. Further studies of MoCAP proteins could lead to a better understanding of the connections between actin organization and host infection by *M*. *oryzae*.

## Introduction

The actin cytoskeleton is a dynamic network critical for various cellular processes in eukaryotic cells, including motility, division, cytokinesis, vesicle trafficking, endocytosis and exocytosis, and cell signaling in response to biotic and abiotic stimuli [1, 2]. Actin dynamics are precisely controlled by a number of regulatory proteins [3, 4], including capping proteins (CAP) [5, 6], actin-regulating kinase (Ark1) [7], and phosphoinositide lipids (PPIs) [8] that associate with and/or regulate the actin cytoskeleton.

The barbed ends of actin filaments play key roles in filament dynamics at cellular structures because of their fast-growing ends for actin polymerization. By binding to the fast barbed ends of actin filaments, the CAP proteins prevent disassembly and addition of new monomers [5, 9]. CAP is a heterodimer protein consisting of alpha (α) and beta (β) subunits that have similar structural folds [10]. CAP proteins are highly conserved in eukaryotic cells, including humans [11], plants [12], and the budding yeast *Saccharomyces cerevisiae* [6]. Studies have shown that null mutants or loss-of-function of CAP result in defects in cellular and developmental processes in mammals, plants, flies, and microbes [13–18]. *CAP* knockdowns in mammalian cells resulted in proliferation of bundled actin in filopodia and loss of lamellipodial arrays at the leading edge of the crawling cells [19]. In *Arabidopsis thaliana*, *CAP* mutants showed abnormal cell morphology but increased actin filament formation [18]. CAP is required for determination of the oocyte and survival of the adult retina in the fly [20, 21]. In *S*. *cerevisiae*, deletion of either *CAP1* or *CAP2* gene induced abnormal F-actin accumulation [13, 21, 22]. The null *CAP* mutants have fewer actin cables but an increased number of actin patches, and the mutants also had growth defect [13, 23].

Also in *S*. *cerevisiae*, Ark1 and Prk1 that is a paralog of Ark1 [7] are serine/threonine protein kinases that affect endocytosis and actin organization [7, 24, 25]. Lack of both Ark1p and Prk1p resulted in the formation of large cytoplasmic actin clumps and severe reduction in growth [7]. The *ark1*Δ*prk1*Δ cells were also defective in the endocytic uptake of the fluorescent fluid-phase marker Lucifer Yellow [25]. Studies have further showed that Ark1 and Prk1 bind to actin cytoskeleton associated proteins and endocytic components to disrupt their activities or their interactions through protein phosphorylation [24–27].

Phosphoinositide lipids (PPIs) are another important component playing a role in regulating the actin cytoskeleton during membrane trafficking [8]. CAP binds to and is negatively regulated by PPIs [28–30]. In *A*. *thaliana*, CAP binds to the signaling lipids phosphatidic acid (PA) and phosphatidylinositol 4,5-bisphosphate (PIP_2_) *in vitro*, which inhibits its barbed end capping activity and causes filament uncapping [31]. Exogenous PA treatment resulted in increased actin filament levels in plant cells [31]. Moreover, the addition of PIP_2_ to a flow chamber with actin filaments that were capped by CAP resulted in the rapid and complete conversion of the actin filaments ends from the non-growing to the growing state [29, 32].

The actin cytoskeleton also plays important roles in plant pathogenic fungi. *Magnaporthe oryzae* is a fungal pathogen that produces appressoria to initiate the infection process and causes the blast disease. Appressoria form actin rings, which are organized mainly by septin to provide cortical rigidity at the initially wall-less region of the appressorium [33]. This actin ring is located at the base of the infection cell surrounding the appressorium pore, a circular region that marks the point where the penetration peg emerges to rupture plant leaf cuticle [33]. The appressorium pore initially lacks a cell wall, and the fungal plasma membrane makes direct contact with the leaf surface of rice (*Oryza sativa* L.). Then, as the appressorium inflates to full turgor pressure, a pore wall overlay develops, and a narrow penetration peg emerges [34]. Assembly of an F-actin network during appressorium turgor generation, just before plant infection, suggests that specific reorientation of the F-actin cytoskeleton takes place at the base of the appressorium to facilitate plant infection. Following penetration, the thin primary penetration hypha differentiates into bulbous and branched infection hyphae [35].

Although the actin cytoskeleton is generally known to play an important role in plant pathogenic fungi, no *CAP* genes have yet been functionally characterized. The relationship between CAP and their regulators such as Ark1 and PIP_2_ was also unknown in these fungi. We here characterized the *MoCAPA* and *MoCAPB* that are important in conidia morphogenesis, appressorial actin ring formation, and infection in *M*. *oryzae*. We provided evidence to demonstrate that MoArk1 regulates MoCAP proteins through protein phosphorylation. In addition, we obtained evidence that PIP_2_ can also regulate CAP proteins function. Our studies provide novel insights into the mechanisms of endocytosis and the actin cytoskeleton and their connections to fungal pathogenicity.

## Results

### Identification of *MoCAPA* and *MoCAPB* and examination of their expression

In the previous study, we found that MoArk1 is an actin-regulating kinase important in the growth, development, and pathogenicity of *M*. *oryzae* [36]. To study how MoArk1 regulates such events, we utilized the affinity purification approach to identify proteins that interact with a tagged MoArk1. We first constructed the *MoARK1*-3xFLAG construct and introduced it into the wild-type stain 70–15 by transformation. Western blot analysis showed the presence of a 117-kDa band, the expected size of the MoArk1-3xFLAG fusion protein in the transformants (S1A Fig). Following affinity purification, proteins bound to the anti-FLAG M2 beads were eluted and analyzed by mass spectrometry (MS). Two proteins encoded respectively by loci MGG_12818.7 and MGG_09902.7 were identified to be homologs to the fungal F-actin CAP protein α and β subunits (S1 Table and S1B and S1D Fig). We named MGG_12818 as *MoCAPA* and MGG_09902 as *MoCAPB*. Further sequence analysis showed that MoCapA contains two conserved F-actin capping motifs, whereas MoCapB contains one F-actin capping motif (S1C and S1E Fig).

To characterize MoCAP proteins, we first profiled the gene transcription at different developmental stages by quantitative real-time PCR (qRT-PCR). The *MoCAP* genes were expressed at all examined stages but had much higher transcription levels at the conidial stage and at 8-h infection stage than at other stages (S2 Fig). The results suggested that MoCAP may play potentially major roles during conidiation and infection stage.

### MoCapA and MoCapB physically interact with MoArk1

To confirm the interactions between two MoCAP proteins and MoArk1, the yeast two-hybrid assay was conducted. The results demonstrated that MoCapA and MoCapB indeed interact with MoArk1 (S3A and S3B Fig).

**Fig 1:**
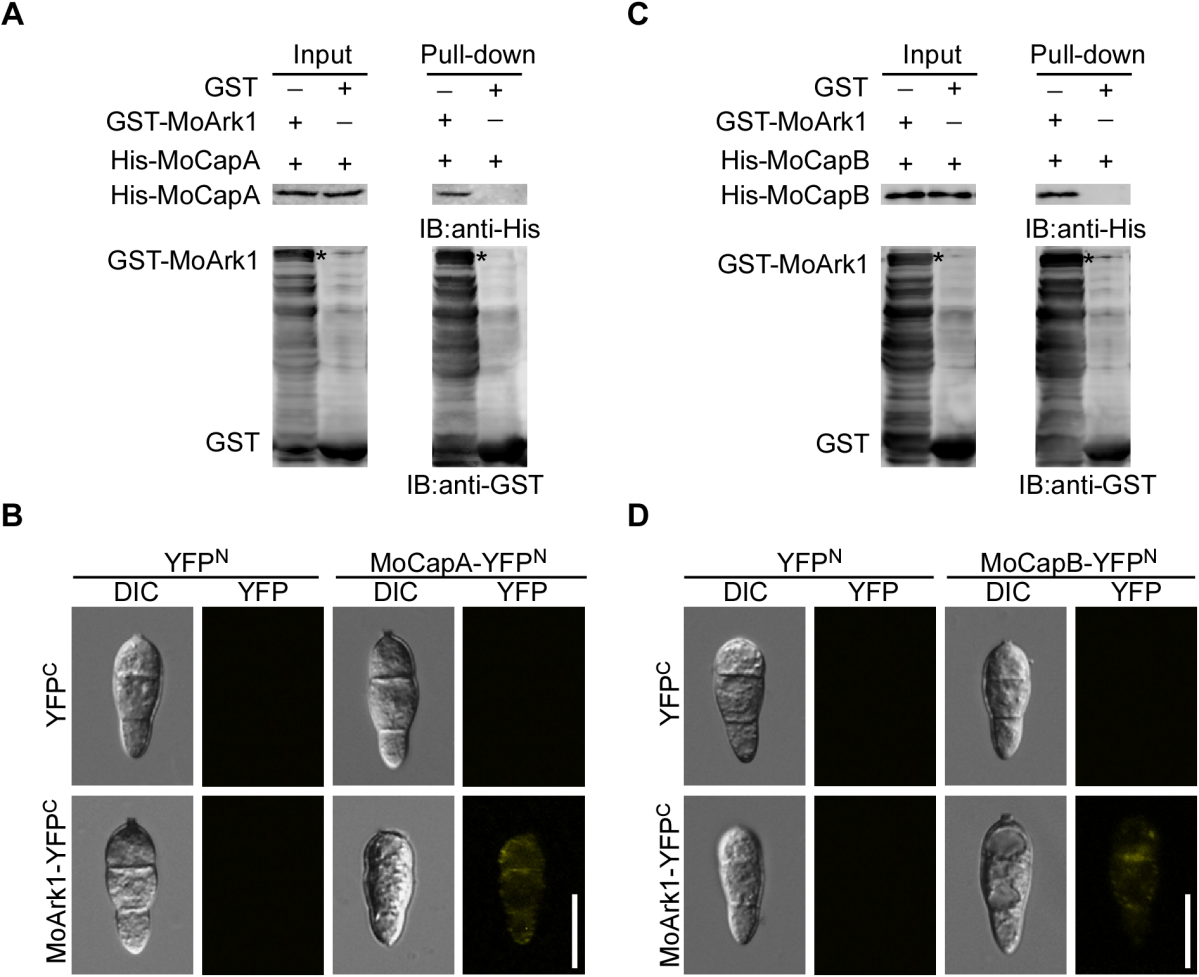
MoCAP proteins interact with MoArk1. (A and C) *In vitro* pull-down assays of MoArk1 and MoCAP proteins. MoArk1-GST- or GST-bound glutathione GST-binding resins were incubated with cell lysates containing MoCapA-His and MoCapB-His, respectively. The precipitation of MoCAP-His with the GST-binding resins was examined by Western blot analysis using the anti-His antibody before (Input) and after wash (Pull-down). The same protein gel was blotted with the anti-GST antibody to show that GST- or GST- MoArk1 was present in the protein mixtures. (B and D) BiFC assays for the MoCAP-MoArk1 interaction. Conidia of transformants with the *MoARK1*-YFP^C^ and *MoCAP*-YFP^N^ constructs were examined by epifluorescence microscopy. Bar = 10 μm.

Additionally, protein pull-down and bimolecular fluorescence complementation (BiFC) assays were employed to validate the interactions. GST-MoArk1, His-MoCapA, and His-MoCapB proteins were expressed in the *Escherichia coli* strain BL21. Whole lysates of GST-MoArk1 or GST were incubated with the whole lysates of His-MoCapA or His-MoCapB, respectively. The mixtures were applied to glutathione GST-binding resin. GST-MoArk1-bound resins, but not GST-bound resins, provided enrichment of His-MoCapA and His-MoCapB (Fig 1A and 1C). Coomassie brilliant blue stained gels also showed the similar results (S3C Fig). For fluorescence complementation, the *MoARK1*-YFP^C^ and *MoCAPA*-YFP^N^, *MoCAPB*-YFP^N^ fusion constructs were generated and introduced into the wild-type strain Guy11 by transformation. YFP signal was observed in the cytoplasm of conidia (Fig 1B and 1D). Collectively, these data indicated that MoCapA and MoCapB physically interact with MoArk1 both *in vitro* and *in vivo*.

### MoCAP proteins are important for polarity growth, conidiation, and full virulence

To functionally characterize MoCapA and MoCapB, targeted gene replacement was performed using the split marker strategy (S4A Fig) [37]. Putative mutants were screened and confirmed by Southern blot analysis (S4B Fig). Three independent Δ*MocapA* and Δ*MocapB* mutants were obtained and all showed similar defective phenotypes. Two double mutant strains were also indistinguishable in general phenotypic characteristics. Therefore, only one mutant strain for either single or double gene disruption was used for further analyses.

**Fig 2:**
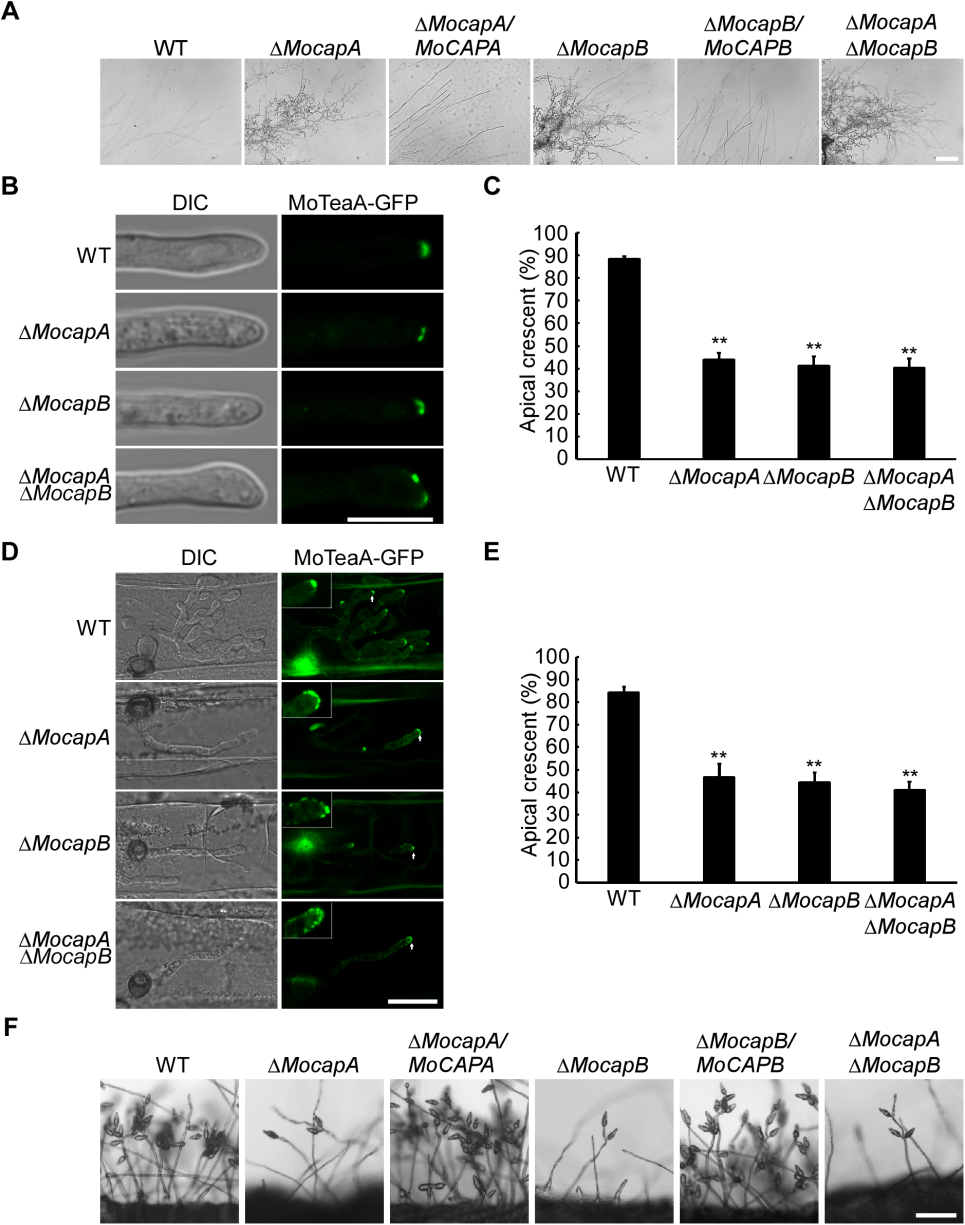
MoCAP proteins are required for polarized growth and conidial production. (A) The hyphae of the Δ*Mocap* mutants are highly branched and curled. The indicated strains were cultured on CM-overlaid microscope slides for 2 days and mycelia were microscopically observed. (B) Location of MoTeaA-GFP in vegetative hyphae. *MoTEAA*-*GFP* expressed under the control of the native promoter in WT, Δ*MocapA*, Δ*MocapB* and Δ*MocapA*Δ*MocapB*. The location of MoTeaA-GFP was microscopically observed following the incubation of WT, Δ*MocapA*, Δ*MocapB* and Δ*MocapA*Δ*MocapB* in liquid CM for 48 h. (C) Bar charts show the percentage of hyphae containing intact apical crescent. The experiment was repeated three times, (**, *p*<0.01), n = 100. (D) Location of MoTeaA-GFP in invasive hyphae. Excised barley (*Hordeum vulgare* cv. Four-arris) leaves from 7-day-old barley seedlings were inoculated with conidial suspension (5 x 10^4^ spores/ml of each strain). The location of MoTeaA-GFP in infectious hyphae was observed at 24 h post-inoculation (hpi). (E) Bar chart to show percentage of infectious hyphae containing intact apical crescent. The experiment was repeated three times, (**, *p*<0.01), n = 100. (F) Conidiation was reduced in the Δ*Mocap* mutants. The indicated strains grown on SDC medium for 7 days were examined by light microscopy. Bar = 5 μm.

For growth assessment, the Δ*Mocap* mutants showed an apparent defect in radial growth (S5A Fig) with altered colony morphology on CM media (S5B Fig). Colony sizes of Δ*MocapA* and Δ*MocapB* were similar, and significantly reduced in comparison with that of the wild-type strain. Similar to the single mutants, the double deletion mutant Δ*MocapA* Δ*MocapB* also exhibited reduced colony formation (S5A and S5B Fig and Table 1). Further, microscopic observation showed that the hyphae of the Δ*Mocap* mutants are highly branched and curled (Fig 2A). And when cultured in liquid CM for 48 h, the mutant strains formed small compact mycelia mass, in contrast to the relatively large mass formed by the wild-type and complemented strains (S5C Fig). We next tested if MoCapA and MoCapB affect the distribution of polarity marker protein MoTeaA. We first examined the localization of MoTeaA in the wild-type background. We found that MoTeaA-GFP occurs symmetrically at an apical crescent of the vegetative hyphae and infected hyphae in wild-type (>80%) as previously seen in *Aspergillus nidulans* [38]. In the Δ*Mocap* mutants, MoTeaA-GFP was present at more than one area in the hyphae and did not concentrate to apical crescent at the apex but localized widely along the infecting hyphal tip cortex (about 58%) (Fig 2B, 2C, 2D and 2E). These results suggested that MoCapA and MoCapB are important for the normal localization of MoTeaA and polarity growth.

**Table 1:** Comparison of mycological characteristics among stains.

	Growth (cm)^α^								Collapsed appressorium rate (%)^β^			
Strain	CM	MM	SDC	OM	Conidiation (×10^4^/cm^2^)^γ^	Abnormal conidia rate (%)	Germination rate(%)^δ^	Appressorium formation(%)^ε^	1 M	2 M	3 M	4 M
WT	4.7±0.1^**A**^	4.1±0.1^**A**^	4.0±0.1^**A**^	4.7±0.1^**A**^	22.5±1.6^**A**^	4.7±1.5^**B**^	95.3±3.5^**A**^	93.7±3.5^**A**^	5.7±1.5^**B**^	24.0±3.0^**B**^	60.1±3.2^**b**^	80.0±2.0^**A**^
Δ*MocapA*	2.1±0.1^**B**^	1.2±0.0^**B**^	1.7±0.1^**B**^	1.9±0.1^**B**^	5.2±0.7^**B**^	40.3±3.1^**A**^	91.7±3.2^**A**^	89.7±2.3^**A**^	13.3±0.58^**A**^	41.7±2.5^**A**^	68.3±2.1^**a**^	84.0±2.0^**A**^
Δ*MocapA/MoCAPA*	4.8±0.1^**A**^	4.0±0.1^**A**^	3.9±0.1^**A**^	4.6±0.1^**A**^	24.1±1.4^**A**^	5.3±1.5^**B**^	93.3±4.0^**A**^	93.0±4.4^**A**^	6.7±1.5^**B**^	25.3±3.1^**B**^	61.7±4.5^**b**^	81.0±1.0^**A**^
Δ*MocapB*	2.0±0.1^**B**^	1.1±0.1^**B**^	1.5±0.2^**B**^	1.8±0.2^**B**^	5.6±0.7^**B**^	43.0±3.6^**A**^	90.7±3.0^**A**^	89.3±3.5^**A**^	14.0±1.7^**A**^	42.7±3.1^**A**^	68.7±1.5^**a**^	84.3±1.5^**A**^
Δ*MocapB/MoCAPB*	4.8±0.2^**A**^	4.0±0.1^**A**^	4.0±0.1^**A**^	4.6±0.1^**A**^	24.1±1.5^**A**^	5.7±1.5^**B**^	94.0±3.0^**A**^	92.7±3.5^**A**^	7.0±1.0^**B**^	26.0±1.7^**B**^	61.0±3.5^**b**^	80.3±2.5^**A**^
Δ*MocapA*Δ*MocapB*	2.0±0.2^**B**^	1.0±0.1^**B**^	1.5±0.2^**B**^	1.8±0.2^**B**^	4.8±1.2^**B**^	43.7±1.5^**A**^	89.7±4.6^**A**^	88.3±4.1^**A**^	15.0±1.7^**A**^	43.3±3.2^**A**^	69.7±3.1^**a**^	84.3±3.8^**A**^

α. Diameter of hyphal radii at day 7 after incubation on CM, MM, SDC and OM agar plates at room temperature.

β. Percentage of collapsed appressoria incubated in 1, 2, 3 and 4 M glycerol solution for 5 min.

γ. Number of conidia harvested from a 9 cm SDC plate at day 10 after incubation at room temperature.

δ. Percentage of conidial germination on artificial surface at 24 h post-inoculation.

ε. Percentage of appressorium formation on artificial surface at 24 h post-inoculation.

The different capital letters in a column show significant difference (*p*<0.01).

The different lower case letters in a column denote significant difference (*p*<0.05).

Microscopic observations showed that conidiation was significantly reduced in the Δ*Mocap* mutants (Fig 2F). On SDC plates at room temperature, the Δ*MocapA* and Δ*MocapB* mutants produced 5.2 × 10^4^/cm^2^ and 5.6 × 10^4^/cm^2^ conidia, respectively, which were significantly less than 22.5 × 10^4^/cm^2^ conidia produced by the wild-type strain. More than 40% of conidia produced by the Δ*Mocap* mutants were abnormal compared with those (<10%) formed by the wild-type strain (S6 Fig and Table 1). In addition, the double deletion mutant was similar (4.8 × 10^4^/cm^2^ conidia) to the single deletion mutants in conidiation (Fig 2F and Table 1).

**Fig 3:**
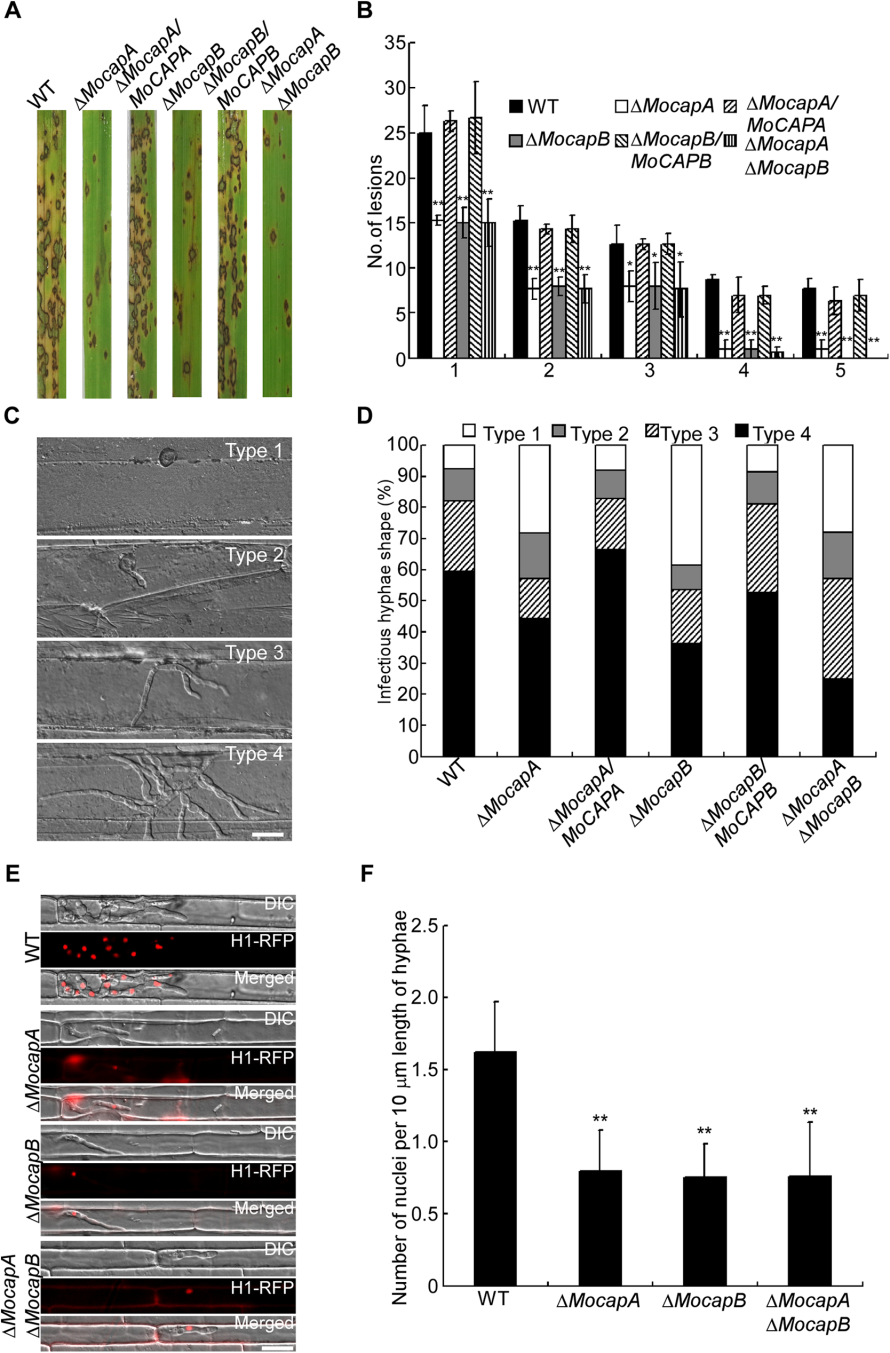
MoCAP proteins are important for full virulence. (A) Rice (*Oryza sativa* cv.CO39) seedlings were inoculated by conidial suspension. Typical leaves were photographed at 7 dpi. (B) Quantification of lesion type (0, no lesion;1, pinhead-sized brown specks; 2, 1.5-mm brown spots; 3, 2–3-mm grey spots with brown margins; 4, many elliptical grey spots longer than 3 mm; 5, coalesced lesions infecting 50% or more of the leaf area). The n means the number of lesions on three rice leaves. Lesions were photographed and measured or scored at 7 days post-inoculation (dpi) and experiments were repeated twice with similar results. Error bars represent the SD and double asterisks indicate statistically significant differences (**, *p*<0.01) and one asterisk represent statistically significant differences (*, *p*<0.05). (C) Close observation of infectious growth on barley. Excised barley leaves from 7-day-old barley seedlings were inoculated with conidial suspension (5 x 10^4^ spores/ml of each strain). Infectious growth was observed at 24 h post-inoculation (hpi). After incubation with the spore suspensions for 24 h, four types (type 1, no penetration; type 2, only with a penetration peg or a single invasive hypha length shorter than 10 μm with no branch; type 3, invasive hyphae length was about 10–20 μm with 1–3 branches; type 4, invasive hyphae length was longer than 20 μm and/or with more than 3 branches) of invasive hyphae were observed in barley tissues [40]. (D) Statistical analysis for each type of infectious hyphal shape, for each tested strain; 100 infecting hyphae (n = 100) were counted per replicate and the experiment was repeated three times. (E) Live-cell imaging at 36 hpi of Guy11 H1:RFP and Δ*Mocap* H1:RFP strains infecting rice leaf sheaths showed Δ*Mocap* H1:RFP strains were impaired in nuclear proliferation in epidermal cells compared to Guy11 H1:RFP. (F) The mean number of nuclei in 10 mm lengths of IH was calculated and the experiment was repeated three times. (**, *p*<0.01), n = 100. Bar = 10 μm.

To assess virulence, conidia of the wild-type strain, Δ*MocapA*, Δ*MocapB* and Δ*MocapA* Δ*MocapB* mutants were inoculated onto rice seedlings at a concentration of 5 × 10^4^ conidia/ml. Few blast lesions were generated by the Δ*Mocap* mutants while the wild-type strain produced a normal number of lesions (Fig 3A). Disease symptoms on the rice leaves were also evaluated using the ‘‘lesion-type” scoring assay [36]. As shown in Fig 3B, the production of type 4–5 and type 1–3 lesions are significantly decreased in the mutants when compared to the wild-type strain (Fig 3B). These results suggested that MoCAP proteins are required for full virulence in the blast fungus.

We then investigated if the attenuation of virulence was due to defects in conidial germination or appressorium formation. However, conidia of the Δ*Mocap* mutants were normal in germination and appressorium formation compared with the wild-type strain (Table 1). To test roles of MoCapA and MoCapB during the infection process more extensively, we conducted infection assays on barley (*Hordeum vulgare* L.) epidermal cells. Again, penetration and invasive growth of the Δ*Mocap* mutants were significantly attenuated. In the wild-type and the complemented strains, type 3 and type 4 invasive hyphae accounted for about 84% with less than 16% being type 1 or type 2 hyphae. In contrast, less than 57% of the hyphae were type 3 and type 4 but more than 40% type 1 and type 2 hyphae were seen in the Δ*Mocap* mutants (Fig 3C and 3D).

To continue gaining the insight into the role of *MoCAP* during the expansion of invasive hyphae in plant cells, we monitored the process by live-cell imaging. A histone H1 protein fused to a red fluorescent protein (H1-RFP) construct was generated and introduced by transformation into protoplasts of the wild-type strain and Δ*Mocap* mutants. After obtaining the positive transformants, we conducted infection assays with rice leaf sheaths. After incubation with the conidial suspensions for 36 h, invasive hyphae were observed in rice leaf sheaths. In the wild-type strain, the intracellular hyphae (IH) contained significantly more nuclei per infected rice cell than the IH of the Δ*Mocap* mutant strains (Fig 3E). The number of nuclei per 10 μm length of mycelia was significantly decreased in mutants compared to wild type (Fig 3F). These results demonstrated that MoCAP proteins are required for expansion of the invasive hyphae during biotrophic colonization of host cells.

Since the turgor pressure within the appressorium is required for successful penetration into host tissue, we measured the appressorial turgor pressure in the Δ*Mocap* mutants using the cytorrhysis assay [39]. Appressoria of the Δ*Mocap* mutants showed an increased collapsing rate in 1–3 M glycerol solutions (Table 1), suggesting that turgor pressure was significantly lower than that in the wild type strain. These results suggested that MoCAP proteins are required for turgor pressure, penetration, and invasive hyphae growth.

### MoCAP proteins are required for normal actin organization

**Fig 4:**
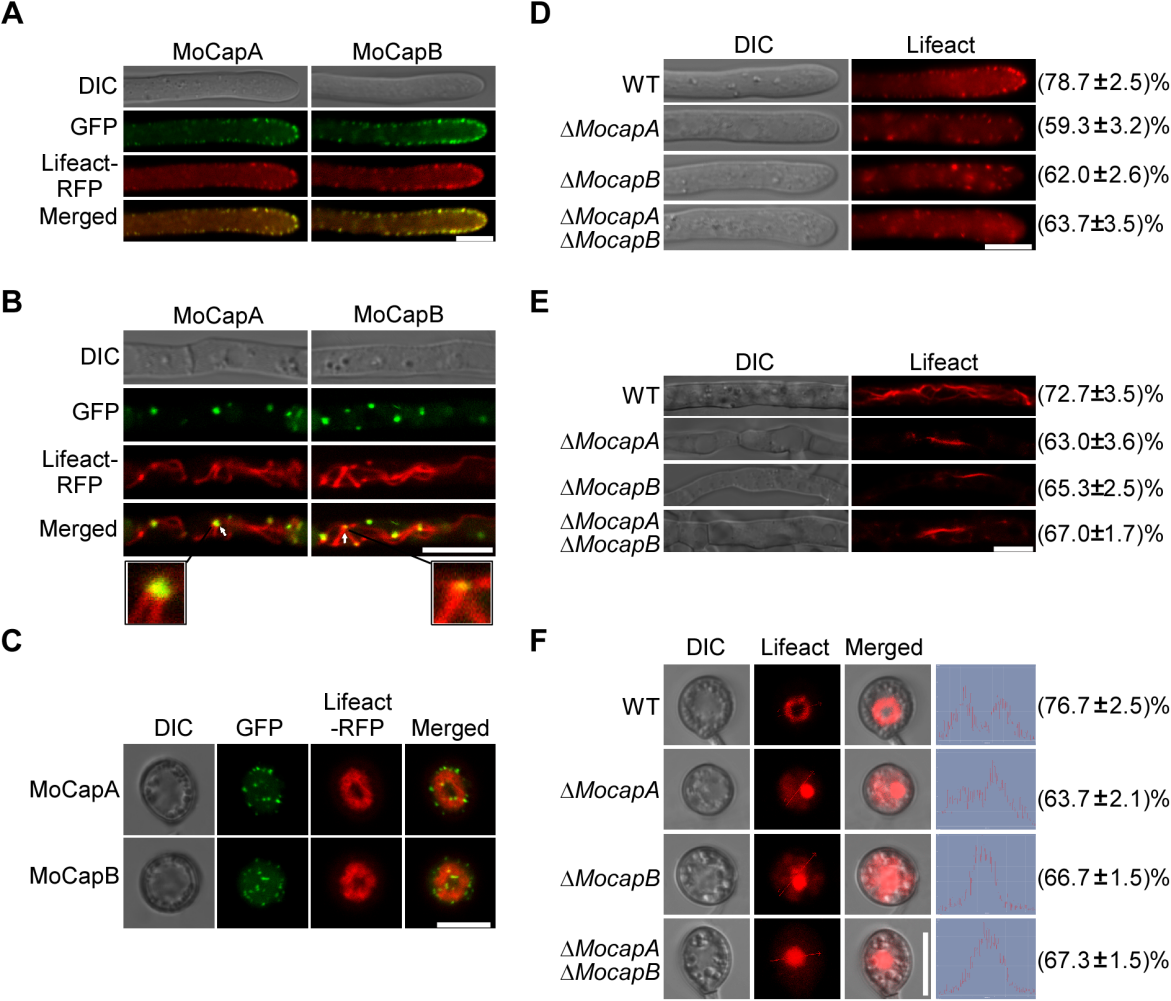
MoCAP proteins are required for normal architecture of actin. (A and B) MoCapA and MoCapB colocalize with the F-actin in vegetative hyphae. The subcellular localization of MoCAP was detected by using a MoCAP-GFP fusion protein driven by its 1.5-kb native promoter. Vegetative hyphae of the transformants expressing the *MoCAPA-GFP* and *MoCAPB-GFP* fusion constructs with the established F-actin marker Lifeact-RFP were observed under laser scanning confocal microscopy (Zeiss LSM710, 63× oil) after incubated in liquid CM for 48 h. (C) Appressoria of the transformants expressing the *MoCAPA-GFP* and *MoCAPB-GFP* fusion constructs with the established F-actin marker Lifeact-RFP were observed after conidia incubated on hydrophobic glass plates for 24 h. (D and E) Actin morphologies in hyphae of WT and mutants. The numbers indicate the percentage of the hyphae exhibiting the actin morphology as seen in figure (n = 100). Micrographs of F-actin were observed by expressing Lifeact-RFP in WT and mutants. Hyphae of the WT and mutants expressing Lifeact-RFP were cultured in liquid CM for 48 h. (F) Actin morphologies in appressoria of WT and mutants. The numbers indicate the percentage of the appressoria exhibiting the actin morphologies as seen in figure (n = 100). Conidial suspensions at 3 × 10^4^ conidia/ml were inoculated onto glass coverslips for 24 h. Bar = 5 μm. The above experiments repeated three times with the same results.

The CAP proteins are key regulators of actin organization and dynamics [5, 9]. In Δ*MocapA/B* expressing *MoCAPA/B-GFP*, fluorescence was detected mainly in patches in vegetative hyphae tips and conidia. To investigate whether MoCAP proteins co-located with F-actin, the *MoCAPA-GFP* and *MoCAPB-GFP* fusion constructs were co-transferred with the actin reporter Lifeact-RFP into the wild-type strain. Most of the MoCapA/B-GFP co-localized with apical cortical patches in the cells of hyphae tip (Fig 4A). And when actin patches appeared in actin cables in the mature cells, MoCapA/B-GFP also partially co-localized with actin patches (Fig 4B). As shown in Fig 4C, MoCapA/B-GFP appeared in the center of the appressoria and surrounded actin ring. To test whether or not loss of CAP affects the actin organization, we performed the actin array of Δ*Mocap* mutants and the wild-type strain expressing the actin reporter, Lifeact-RFP. The actin patches close to the cytomembrane were reduced in the vegetative hyphae tip of the Δ*Mocap* mutants compared with wild-type (Fig 4D). In wild-type strains, the RFP signal increased in the apical membrane of hyphae and formed apical cortical patches (about 78%) (Fig 4D and S1 Video). There were significantly reduced apical cortical patches at the top of the Δ*Mocap* mutant hyphae. About 60% the Δ*Mocap* mutant hyphae did not exhibit obvious apical cortical patches (Fig 4D and S2, S3 and S4 Videos). Most wild-type strains (about 72%) generally showed several cables in the mature cells (Fig 4E). In contrast, the Δ*Mocap* mutants only formed cables that are less numerous, less intense, and shorter in the mature cells (about 63%) (Fig 4E). Notably, in appressorium formed by the wild type strain, an actin ring was observed at the base of the infection cell (76.7%) surrounding the appressorium pore (Fig 4F). While in the appressoria of Δ*Mocap*, instead of the regular actin rings, condensed Lifeact RFP ball-like structures (about 63%) were observed (Fig 4F). These data indicated that MoCAP proteins are required for actin organization both in vegetative hyphae and in appressorium.

### MoCAP proteins bind with actin through F-actin-capping motifs

**Fig 5:**
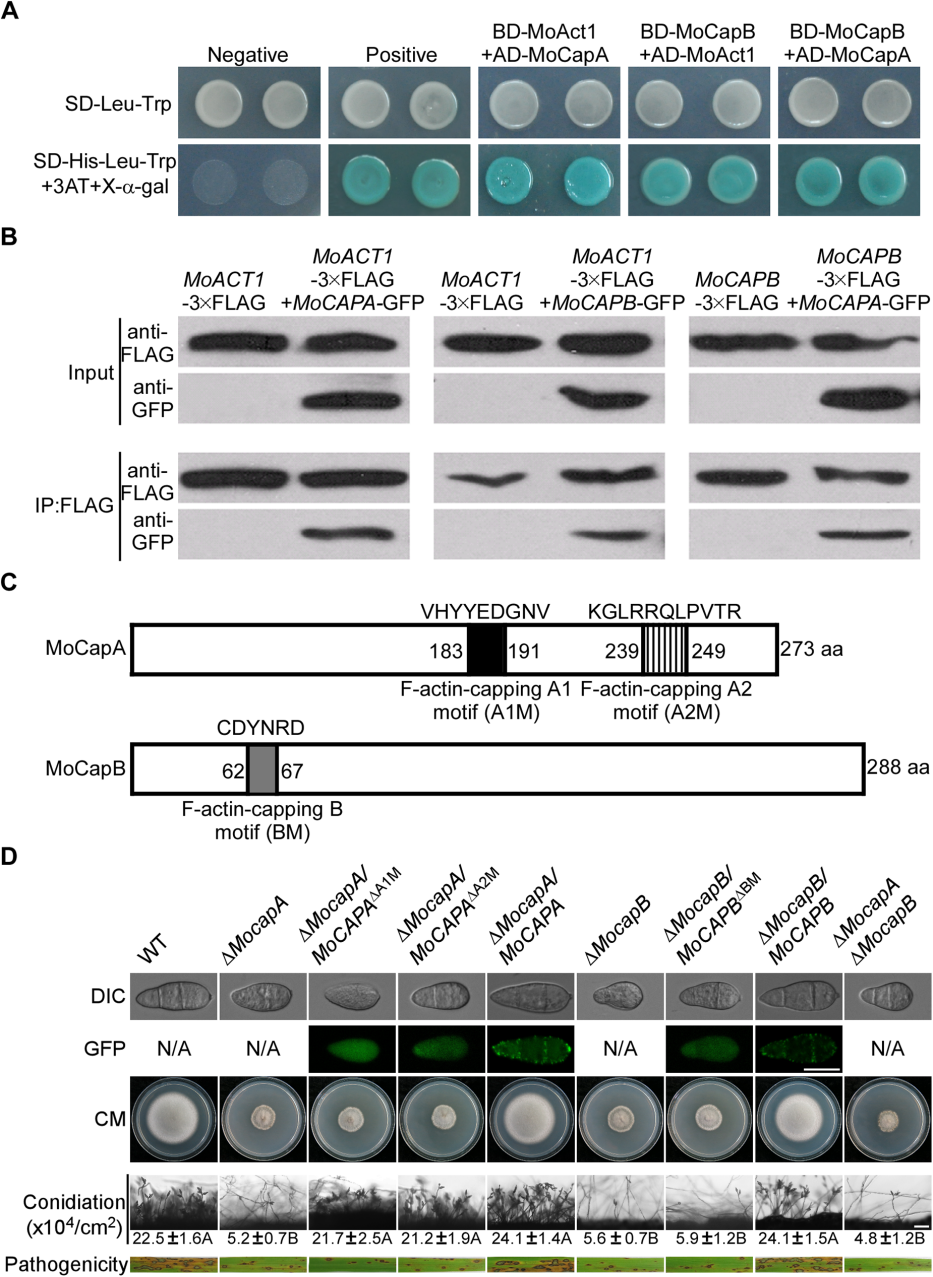
F-actin-capping motifs are required for the localization and functions of MoCAP proteins. (A) Yeast two-hybrid assay for the interaction between MoCapA, MoCapB and MoAct1. Yeast transformants expressing the prey and bait constructs were assayed for growth on SD-Leu-Trp and SD-Leu-Trp-His plates and β-galactosidase activities (LacZ). (B) Co-immunoprecipitation (CoIP) analyses of interaction between MoCapA, MoCapB and MoAct1. The *MoACT1*-3xFLAG/*MoCAPA*-GFP, *MoACT1*-3xFLAG/*MoCAPB*-GFP, and *MoCAPA*-3xFLAG/*MoCAPB*-GFP were co-expressed in the wild-type strain, respectively. The Co-IP experiment was performed with the anti-GFP antibody, and the isolated protein was analyzed by western blot using anti-FLAG and anti-GFP antibodies. (C) F-actin-capping motifs of MoCAP proteins were analyzed by motif scan. (D) Subcellular localization of motif-deleted proteins and growth, conidiation and pathogenicity of the motif deletion mutants (Δ*MocapA/MoCAPA*^ΔA1M^, Δ*MocapA/MoCAPA*^ΔA2M^ and Δ*MocapB/MoCAPB*^ΔBM^). All strains were cultured on complete medium (CM) following incubation of plates at 28°C for 7 days in the dark. Disease symptoms on rice seedlings sprayed with conidial suspensions at 7 dpi. Bar = 10 μm.

CAP proteins bind to barbed actin filament ends with high affinity, thereby blocking actin assembly and disassembly [32]. To test whether MoCapA and MoCapB directly interact with the actin protein MoAct1, we employed the yeast two-hybrid assay and constructed BD-MoAct1, AD-MoCapA, AD-MoAct1, and BD-MoCapB. The final results showed that both subunits of MoCAP not only directly interact with MoAct1, but also each other (Fig 5A).

To examine whether the interactions between MoAct1, MoCapA or MoCapB occur *in vivo*, the *MoACT1*-3xFLAG, *MoCAPA*-3xFLAG, *MoCAPA*-GFP and *MoCAPB*-GFP fusion constructs were generated and introduced by co-transformation in various combinations into the wild-type strain. Total protein was isolated from the positive transformants and western blot analysis was performed. The anti-FLAG and anti-GFP antibodies detected the presence of a 43- and a 57-kDa band, respectively. In proteins eluted from anti-FLAG M2 beads, the 57-kDa MoCapA-GFP band was detected in the transformant expressing the *MoCAPB*-3xFLAG construct and *MoCAPA*-GFP construct, but was not in the transformant expressing the *MoCAPB*-3xFLAG construct only (Fig 5B). Similar methods were used to test the transformant co-expressing *MoACT1*-3xFLAG with *MoCAPA*-GFP or *MoCAPB*-GFP fusion constructs (Fig 5B). These results suggest that MoCapA and MoCapB interact with MoAct1 while MoCapA interacts with MoCapB.

Bioinformatics analyses suggest that MoCAP proteins contain the F-actin-capping motifs (S1 Fig). To further assess the role of the motifs, we obtained the *MoCAPA*^ΔA1M^-GFP and *MoCAPA*^ΔA2M^-GFP transformants (Fig 5C). The Δ*MocapA/MoCAPA*^ΔA1M^-GFP transformant expressing *MoCAPA* lacking the F-actin-capping A1 motif and the Δ*MocapA/MoCAPA*^ΔA2M^-GFP transformant lacking the F-actin-capping A2 motif were produced. In these transformants, GFP fluorescence appeared in the cytoplasm of conidia with no actin localization pattern shown. However, defects in growth and pathogenicity were similar to those of the Δ*MocapA* mutant, except for the production of conidia that was similar to the wild-type strain (Fig 5D). A Δ*MocapB/MoCAPB*^ΔBM^-GFP transformant, expressing *MoCAPB* without the F-actin-capping B motif, was also produced. This transformant showed dispersed GFP signal in the cytoplasm, compared with the Δ*MocapB/MoCAPB*-GFP transformant. The *MoCAPB*^ΔBM^-GFP could not suppress the defects in growth, conidiation, or pathogenicity of the Δ*MocapB* mutant (Fig 5D).

Finally, motif deletion analysis indicated that the F-actin-capping motifs are responsible for the subcellular localization of MoCAP proteins, which colocalize with actin and directly interact with MoAct1. To test if F-actin-capping motifs are involved in the interactions between MoCapA/B and MoAct1, the yeast two-hybrid assay was performed using the AD-*MoCAPA*^ΔA1M^, AD-*MoCAPA*^ΔA2M^, AD-*MoCAPB*^ΔBM^ and BD-*MoACT1* constructs. The results showed that all construct combinations did not confer any growth for the yeast strain AH109 on selective medium (S7 Fig), indicating that MoAct1 did not interact with MoCapA^ΔA1M^, MoCapA^ΔA2M^ or MoCapB^ΔBM^. Thus, the F-actin-capping motifs are essential for the interactions between MoCAP and MoAct1.

Taken together, the above results indicated that the capping motifs are responsible for subcellular localization of MoCAP proteins, as well as growth, conidiation and pathogenicity of *M*. *oryzae*.

### MoCAP proteins are involved in endocytosis

**Fig 6:**
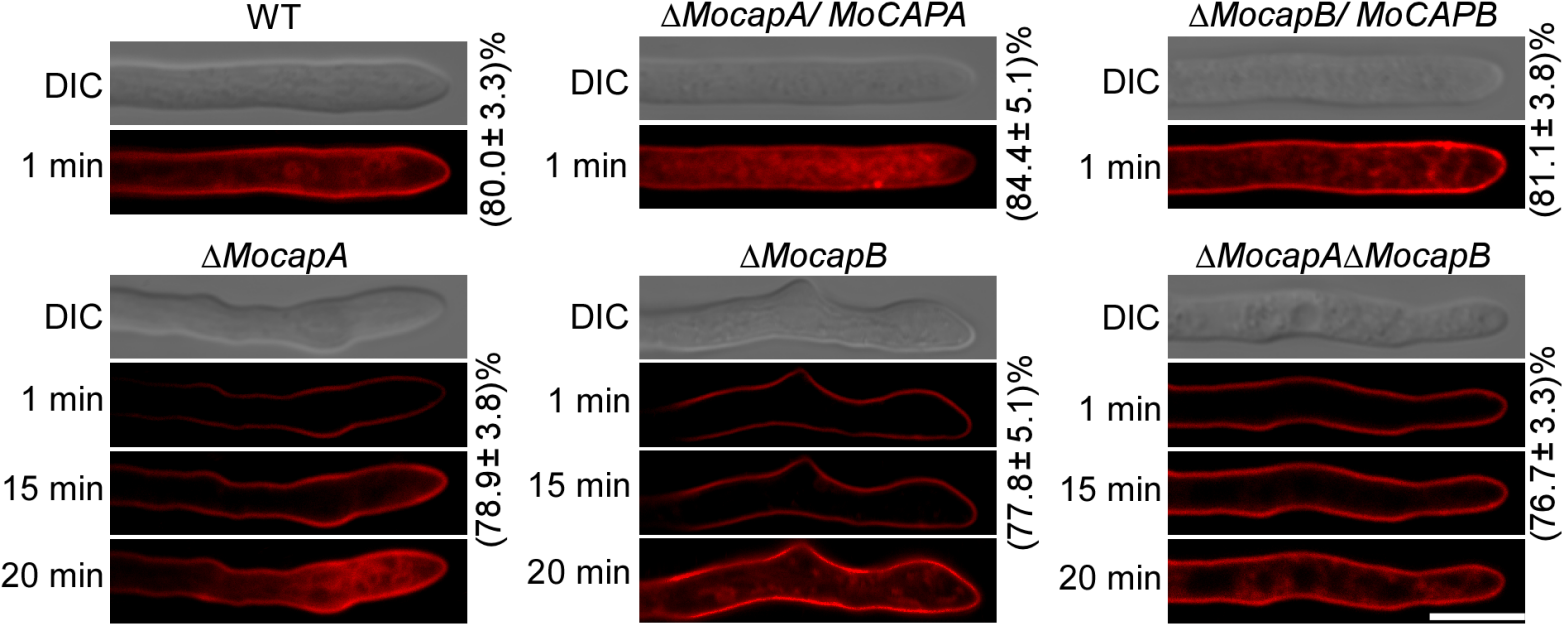
MoCAP proteins are important for normal endocytosis. The numbers indicate the percentage of the hyphae exhibiting different endocytosis as seen in figure (n = 100). Strains were grown in liquid CM for 48 h before the addition of FM4-64 and photographs were taken under confocal fluorescence microscope (Zeiss LSM710, 63 × oil) after different time of exposure to FM4-64. Camera exposure is indicated in seconds (800 ms). Bar = 5 μm. The experiment repeated three times with the same results.

Previously, the loss of *MoARK1* resulted in defects in endocytosis and other intracellular transport in *M*. *oryzae* [36]. Because MoCapA and MoCapB were shown to interact with MoArk1, we further tested whether MoCAP proteins are also required for these processes. Within 1 min about 80% of wild-type cells and the complementary transformants stained with FM4-64, signal appeared on the plasma membrane and endomembrane compartments, such as vacuoles and endosomes (Fig 6). However, the internalization of FM4-64 dye did not occur until 15 min after staining in the mutant cells. When stained for 20 min, similar to the wild-type strain, clear vacuolar fluorescence signal was observed (about 76%) in the Δ*Mocap* mutant cells (Fig 6). These results indicated that MoCAP proteins are required for normal endocytosis process.

### MoCapA and MoCapB are phosphorylated substrates of MoArk1

**Fig 7:**
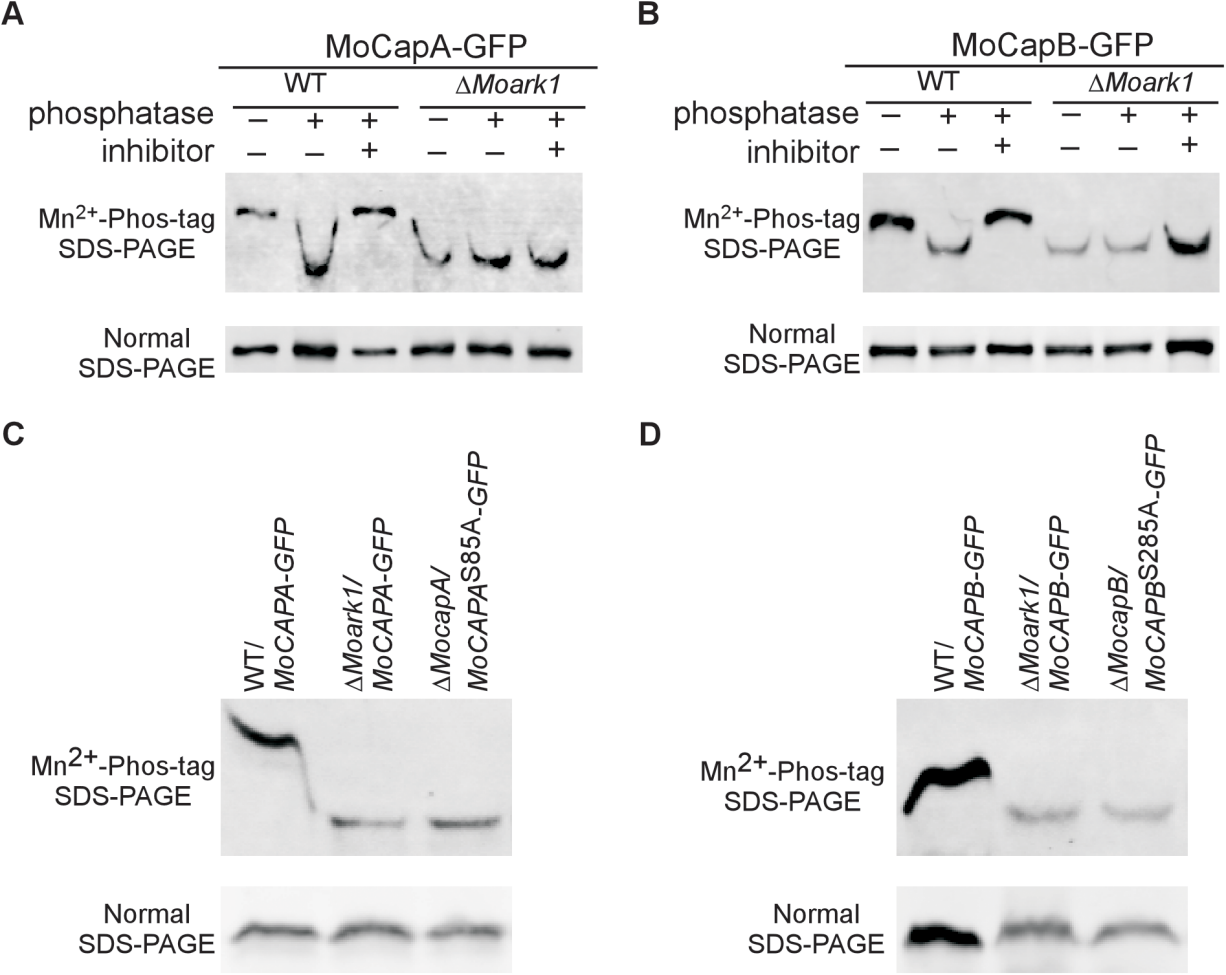
MoCapA and MoCapB are phosphorylated by MoArk1. (A and B) Green fluorescent protein-MoCapA (MoCapA-GFP) and green fluorescent protein-MoCapB (MoCapB-GFP) from wild-type and the Δ*Moark1* mutant cell extracts treated with alkaline phosphatase or alkaline phosphatase and phosphatase inhibitors were subjected to Phos-tag SDS-PAGE and normal SDS-PAGE followed by immunoblotting with anti-GFP. (C and D) As MoCapA-GFP and MoCapB-GFP from wild-type and the Δ*Moark1* mutant cell extracts were contrast, the phosphorylation of MoCapA^S85A^ and MoCapB^S285A^ was examined applying the Phos-tag SDS-PAGE method. The mobility shift of MoCapA^S85A^ and MoCapB^S285A^ is same to the MoCapA and MoCapB in Δ*Moark1* but not the wild type.

In mammalian and yeast cells, the Ark and Prk serine/threonine kinases initiate phosphorylation cycles to control the endocytic machinery and the actin cytoskeleton [41]. Since MoArk1 interacts with MoCapA and MoCapB, we tested whether MoArk1 phosphorylates the MoCAP proteins. The *MoCAPA-GFP* and *MoCAPB-GFP* constructs were transferred respectively into the wild-type strain and Δ*Moark1* mutant. We analyzed the MoCapA-GFP and MoCapB-GFP from the wild-type strain and Δ*Moark1* mutant on gels containing the Phos-tag, which retards the mobility of phosphoproteins [42]. Whole-cell extracts were treated either with phosphatase or phosphatase inhibitor, and mobility shifts were examined by immunoblotting with the anti-GFP antibody. The reduced mobility form of MoCapA-GFP and MoCapB-GFP was present in untreated and phosphatase and phosphatase inhibitor treated wild-type cells but not in the phosphatase treated wild-type cells. The similar phosphatase-sensitive and slow moving band was not observed in extracts from untreated Δ*Moark1* mutant cells (Fig 7A and 7B). The decreased mobility of MoCapA-GFP and MoCapB-GFP of the untreated wild-type strain compared to untreated Δ*Moark1* mutant indicated the higher level of phosphorylation of MoCapA-GFP and MoCapB-GFP in the wild type strain. These results suggested that MoArk1 could regulate MoCAP proteins through protein phosphorylation.

To identify phosphorylation sites on MoCapA or MoCapB, we purified the fusion proteins of MoCapA and MoCapB with the green fluorescent protein (MoCapA-GFP and MoCapB-GFP) (S8 Fig). Mass spectrometry showed that the serine at the 85th residue (S85) of MoCapA and serine at the 285th residue (S285) of MoCapB were phosphorylated (S9 Fig). To further verify these phosphorylation sites, we expressed the *MoCAPA*^S85A^-*GFP* construct in Δ*MocapA* and the *MoCAPB*^S285A^-*GFP* construct in Δ*MocapB*, respectively, and obtained the Δ*MocapA*/*MoCAPA*^S85A^-*GFP* and Δ*MocapB*/*MoCAPB*^S285A^-*GFP* transformants. We then examined the phosphorylation of MoCapA^S85A^ and MoCapB^S285A^ employing the Phos-tag SDS-PAGE method as mentioned above. Indeed, the mobility shifts of MoCapA^S85A^ and MoCapB^S285A^ were the same as the MoCapA and MoCapB in Δ*Moark1*, but not wild type (Fig 7C and 7D). These data indicated that MoArk1 phosphorylates MoCapA on the 85th serine and MoCapB on the 285th serine.

### The phosphorylation at MoCapA Ser-85 and MoCapB Ser-285 is inhibitory to MoCAP proteins functions

**Fig 8:**
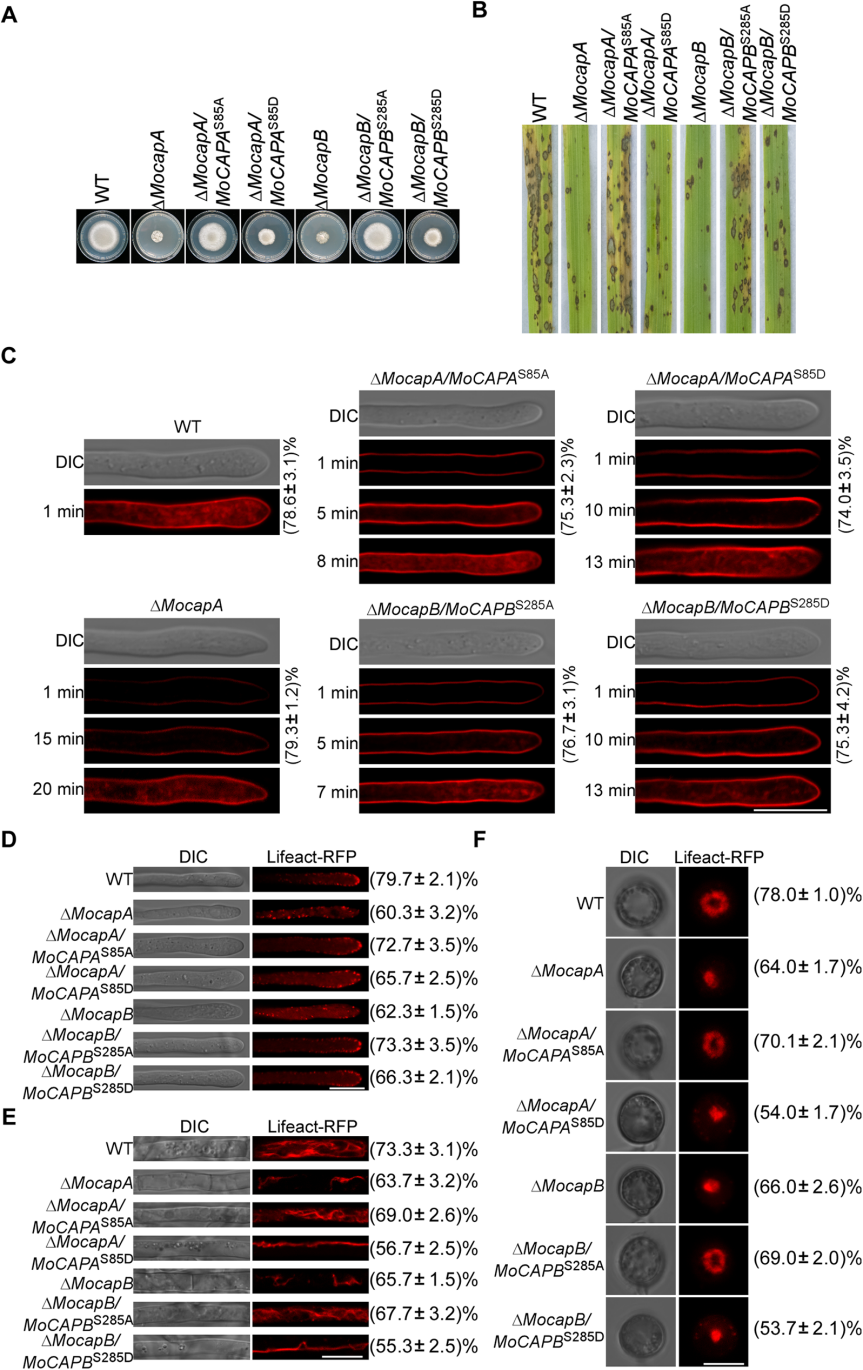
The phosphorylation of MoCapA and MoCapB negatively regulate growth, virulence, endocytosis and F-actin assembly. (A) Growth of WT, Δ*MocapA*, Δ*MocapA/MoCAPA*^S85A^, Δ*MocapA/MoCAPA*^S85D^, Δ*MocapB*, Δ*MocapB/MoCAPB*^S285A^ and Δ*MocapB/MoCAPB*^S285D^. Seven-day-old cultures of different strains on CM plates. (B) Pathogenicity of WT, Δ*MocapA*, Δ*MocapA/MoCAPA*^S85A^, Δ*MocapA/MoCAPA*^S85D^, Δ*MocapB*, Δ*MocapB/MoCAPB*^S285A^ and Δ*MocapB/MoCAPB*^S285D^. Disease symptoms on rice seedlings sprayed with conidial suspensions at 7 dpi. (C) The effect of phosphorylation of MoCAP on endocytosis. The numbers indicate the percentage of the hyphae exhibiting different endocytosis as seen in figure (n = 100). Strains were cultured in liquid CM for 48 h, then stained with FM4-64 and examined under confocal fluorescence microscope (Zeiss LSM710, 63×oil). The camera exposure is indicated in seconds (800 ms). Here Δ*MocapA* represents all Δ*Mocap* mutants as they exhibit the similar defect in endocytosis as seen in Fig 6. (D and E) Actin morphologies in hyphae were observed after strains were cultured in liquid CM for 48 h. The numbers indicate the percentage of the hyphae exhibiting actin morphologies as seen in figure (n = 100). (F) Actin morphologies in appressoria were observed. The numbers indicate the percentage of the appressoria exhibiting actin morphologies as seen in figure (n = 100). Observations were made with a confocal fluorescence microscope (Zeiss LSM710, 63×oil). Bar = 5 μm. The above experiments repeated three times with the same results.

The effect of phosphorylation site mutations were further determined. Converting serines to either asparagines or alanines mimics constitutively phosphorylated or dephosphorylated MoCapA and MoCapB, respectively (*MoCAPA*^S85D^, *MoCAPB*^S285D^ and *MoCAPA*^S85A^, *MoCAPB*^S285A^). The constructs of constitutively phosphorylated and dephosphorylated *MoCAPA* and *MoCAPB* were introduced into Δ*MocapA* and Δ*MocapB*, respectively (S10 Fig). The growth, spore-forming and virulence defects were partially suppressed in all the phosphorylation site mutation alleles. Constitutively phosphorylated mutants all exhibit faster growth, higher spore yield and stronger virulence than the Δ*MocapA* and Δ*MocapB* mutants but still slower, lower and weaker than WT (Fig 8A and 8B and S2 Table). Constitutively dephosphorylated mutants also showed faster growth and higher conidiation than Δ*Mocap* mutants but still slower and lower than WT, whereas maintained almost identical virulence as of to the wild type. However, we also found that constitutively phosphorylated mutants showed more serious defects compared with the constitutively dephosphorylated mutants. The Δ*MocapA*/*MoCAPA*^S85D^ and Δ*MocapB*/*MoCAPB*^S285D^ transformants exhibit poorer growth, less conidial production, and lower virulence than the Δ*MocapA*/*MoCAPA*^S85A^ and Δ*MocapB*/*MoCAPB*^S285A^ transformants (Fig 8A and 8B and S2 Table). These results suggested that the constitutively phosphorylated MoCAP proteins may be inhibitory to their own function.

Next, we further tested effects of constitutively phosphorylated and dephosphorylated MoCapA and MoCapB on endocytosis. The endocytic function was assessed with FM 4–64 uptake. All of the mutations in the phosphorylation site can partly compensate the endocytosis defect of Δ*Mocap* mutants. As previously described, within 1 min wild-type cells stained with FM4-64, signal appeared on the plasma membrane and endomembrane compartments (about 78%), such as vacuoles and endosomes (Fig 8C). The internalization of FM4-64 dye did not occur until 8 min (Δ*MocapA/MoCAPA*^S85A^, about 75%) and 7 min (Δ*MocapB/MoCAPB*^S285A^, about 76%) after staining in the sustained dephosphorylation mutant cells. However, until staining for 13 min, the vacuolar fluorescence signal was observed in the continuously phosphorylation mutant cells (Δ*MocapA/MoCAPA*^S85D^ and Δ*MocapB/MoCAPB*^S285D^, about 74% and 75% respectively). These results indicated that the phosphorylation of MoCapA and MoCapB by MoArk1 negatively regulates endocytosis.

To test further whether or not phosphorylation of MoCAP affects the actin organization in *M*. *oryzae*, the F-actin marker Lifeact-RFP was introduced into the constitutively phosphorylated and dephosphorylated mutants. We found that the phosphorylation site mutants (constitutively dephosphorylated mutants, about 72% and constitutively phosphorylated mutants, about 65%) can form apical cortical patches as seen in wild-type strains (Fig 8D). In the mature cells, similar to wild-type the constitutively dephosphorylated mutants (about 67%) can form relatively more actin cables than the constitutively phosphorylated and knockout mutant. The actin cables in sustained phosphorylation mutants (about 55%) were longer than Δ*Mocap* mutants (Fig 8E). In appressorium, an actin ring was observed at the base of the infection cell surrounding the appressorium pore in constitutively dephosphorylated mutants (>69%) as similar to the wild-type. While in about 53% of the appressoria of constitutively phosphorylated mutants, similar to knockout mutants, condensed Lifeact RFP ball-like structures were observed (Fig 8F). These data indicated that the phosphorylation of MoCAP proteins negatively regulate the actin organization both in vegetative hyphae and in appressorium.

### Constitutively dephosphorylated MoCapA and MoCapB can suppress Δ*Moark1* defects

**Fig 9:**
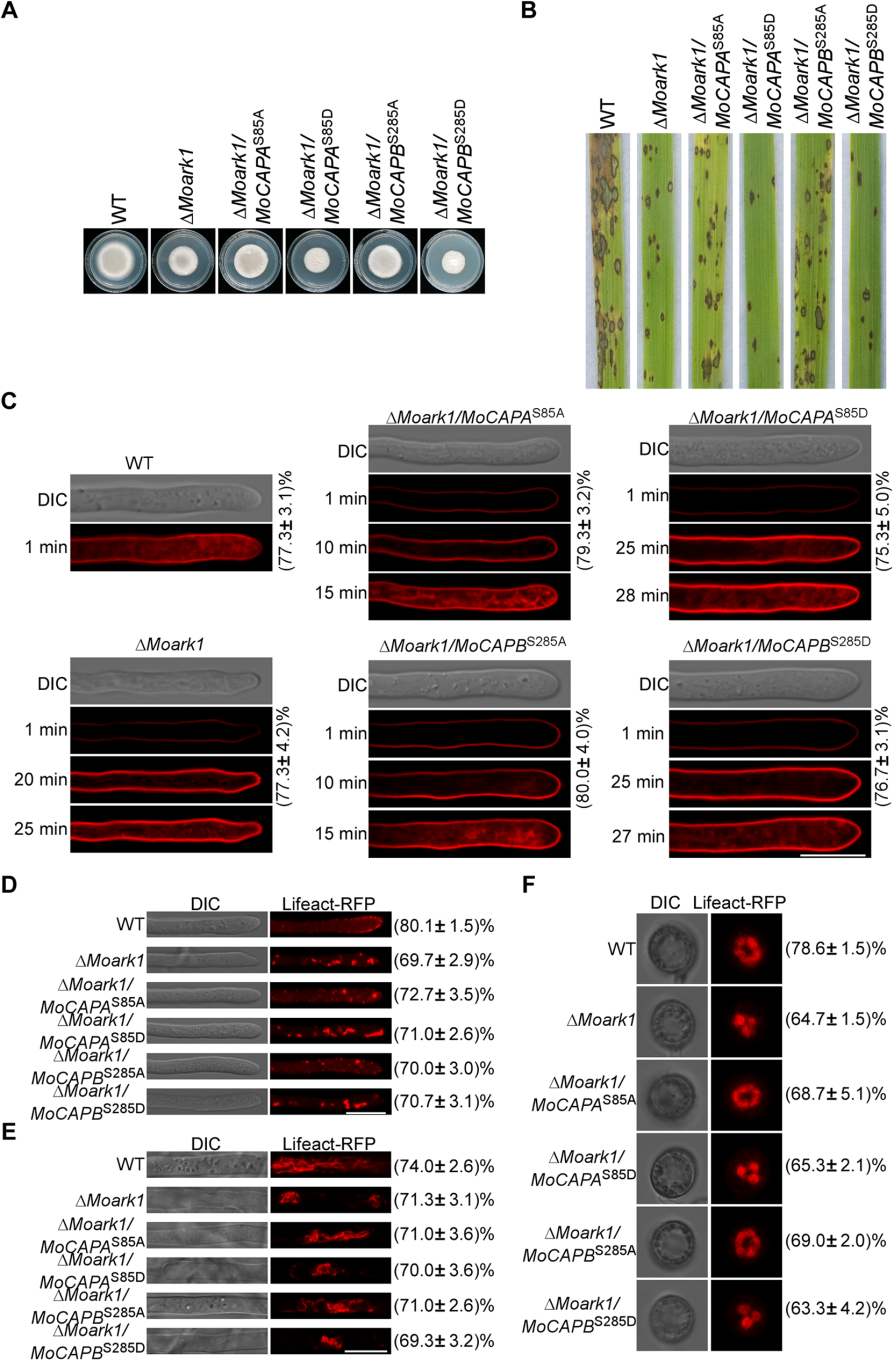
Constitutively dephosphorylated MoCapA and MoCapB can suppress Δ*Moark1* defects. (A) Growth of WT, Δ*Moark1*, Δ*Moark1/MoCAPA*^S85A^, Δ*Moark1/MoCAPA*^S85D^, Δ*Moark1/MoCAPB*^S285A^ and Δ*Moark1/MoCAPB*^S285D^. Seven-day-old cultures of different strains on CM plates. (B) Pathogenicity of WT, Δ*Moark1*, Δ*Moark1/MoCAPA*^S85A^, Δ*Moark1/MoCAPA*^S85D^, Δ*Moark1/MoCAPB*^S285A^ and Δ*Moark1/MoCAPB*^S285D^. Disease symptoms on rice seedlings sprayed with conidial suspensions at 7 dpi. (C) The effect of phosphorylation of MoCAP on endocytosis defects of the Δ*Moark1* mutant. The numbers indicate the percentage of the hyphae exhibiting different endocytosis as seen in figure (n = 100). Strains were cultured in liquid CM for 48 h, then stained with FM4-64 and examined under confocal fluorescence microscope (Zeiss LSM710, 63×oil). Camera exposure is indicated in seconds (800 ms). (D and E) Actin morphology in hyphae. Observations were made after the indicated strains were cultured in liquid CM for 48 h. The numbers indicate the percentage of the hyphae exhibiting actin morphologies as seen in figure (n = 100). (F) Actin morphologies in appressoria of indicated strains. The numbers indicate the percentage of the appressoria showing actin morphologies as seen in figure (n = 100). Observations were made with a confocal fluorescence microscope (Zeiss LSM710, 63×oil). Bar = 5 μm.

In the previous studies, the *MoARK1* deletion mutants exhibited poor growth, low virulence and severe endocytosis defects in *M*. *oryzae* [36]. Our biochemical data indicate that MoArk1 is required for MoCapA and MoCapB serine phosphorylation. In order to test the effects of continuously phosphorylated and dephosphorylated MoCapA and MoCapB on Δ*Moark1*, the constitutively phosphorylated and dephosphorylated *MoCAPA* and *MoCAPB* constructs were introduced into Δ*Moark1*, respectively (Δ*Moark1*/*MoCAPA*^S85D^, Δ*Moark1*/*MoCAPA*^S85A^, Δ*Moark1*/*MoCAPB*^S285D^ and Δ*Moark1*/*MoCAPB*^S285A^) (S11 Fig). As shown in Fig 9, *MoCAPA*^S85A^ and *MoCAPB*^S285A^ were able to partially suppress the defects in growth, sporulation capacity and pathogenicity. However, *MoCAPA*^S85D^ and *MoCAPB*^S285D^ could not suppress these defects, and in fact aggravated these defects (Fig 9A and 9B and S3 Table). These results indicated that MoCAP proteins are the targets of MoArk1 regulation and revealed a surprisingly specific property of MoCapA and MoCapB for the suppression of Δ*Moark1* phenotypes.

In order to test effects of constitutively phosphorylated and dephosphorylated MoCapA and MoCapB on endocytosis defects of the Δ*Moark1* mutant, we performed FM 4–64 dye uptake experiments. As seen in the previous study, the Δ*Moark1* exhibited defects in FM 4–64 uptake and the defect can be suppressed by expressing constitutively dephosphorylated MoCapA and MoCapB. However the defect was aggravated by expressing constitutively phosphorylated MoCapA and MoCapB. The Δ*Moark1*/*MoCAPA*^S85A^ (79%) and Δ*Moark1*/*MoCAPB*^S285A^ (80%) strains exhibited relatively faster FM4-64 uptake (15 min) than Δ*Moark1* (25 min). Δ*Moark1*/*MoCAPA*^S85D^ (75%) and Δ*Moark1*/*MoCAPB*^S285D^ (76%) were slower in FM 4–64 uptake (Δ*Moark1*/*MoCAPA*^S85D^ 28 min and Δ*Moark1*/*MoCAPB*^S285D^ 27 min) than Δ*Moark1* (25 min) (Fig 9C).

The actin regulating kinase Ark1 is very important to regulate actin cytoskeleton in yeast [27, 43]. In this study, we also explored the effect of *MoARK1* absence on the actin cytoskeleton. As shown in Fig 9, instead of forming apical cortical patches, as seen in the hypha tip cells of wild-type strains, the Lifeact-RFP signal was mainly observed in large clumps in Δ*Moark1* (69%). In mature hyphae cells, actin cables of Δ*Moark1* (71%) assembled in disordered clusters compared with the ordered filamentous actin cables of the wild type. During appressoria, actin in Δ*Moark1* mutant (64%) did not form ring but accumulated in a number of clumps. We further examined the effects of constitutively phosphorylated and dephosphorylated MoCapA and MoCapB on the actin defects of Δ*Moark1*. As seen in Fig 9D, 9E and 9F, constitutively dephosphorylated MoCapA (>68%) and MoCapB (>69%) were able to partially suppress the accumulation of actin clumps in Δ*Moark1*. But this partial suppression of actin clump accumulation did not occur in Δ*Moark1* expressing constitutively phosphorylated MoCapA and MoCapB (Δ*Moark1*/*MoCAPA*^S85D^, >65% and Δ*Moark1*/*MoCAPB*^S285D^, >63%). These results furthermore suggested that the phosphorylation of MoCapA and MoCapB mediated by MoArk1 inhibits MoCapA and MoCapB function.

### MoCAP proteins regulate the actin organization in response to PIP_2_

**Fig 10:**
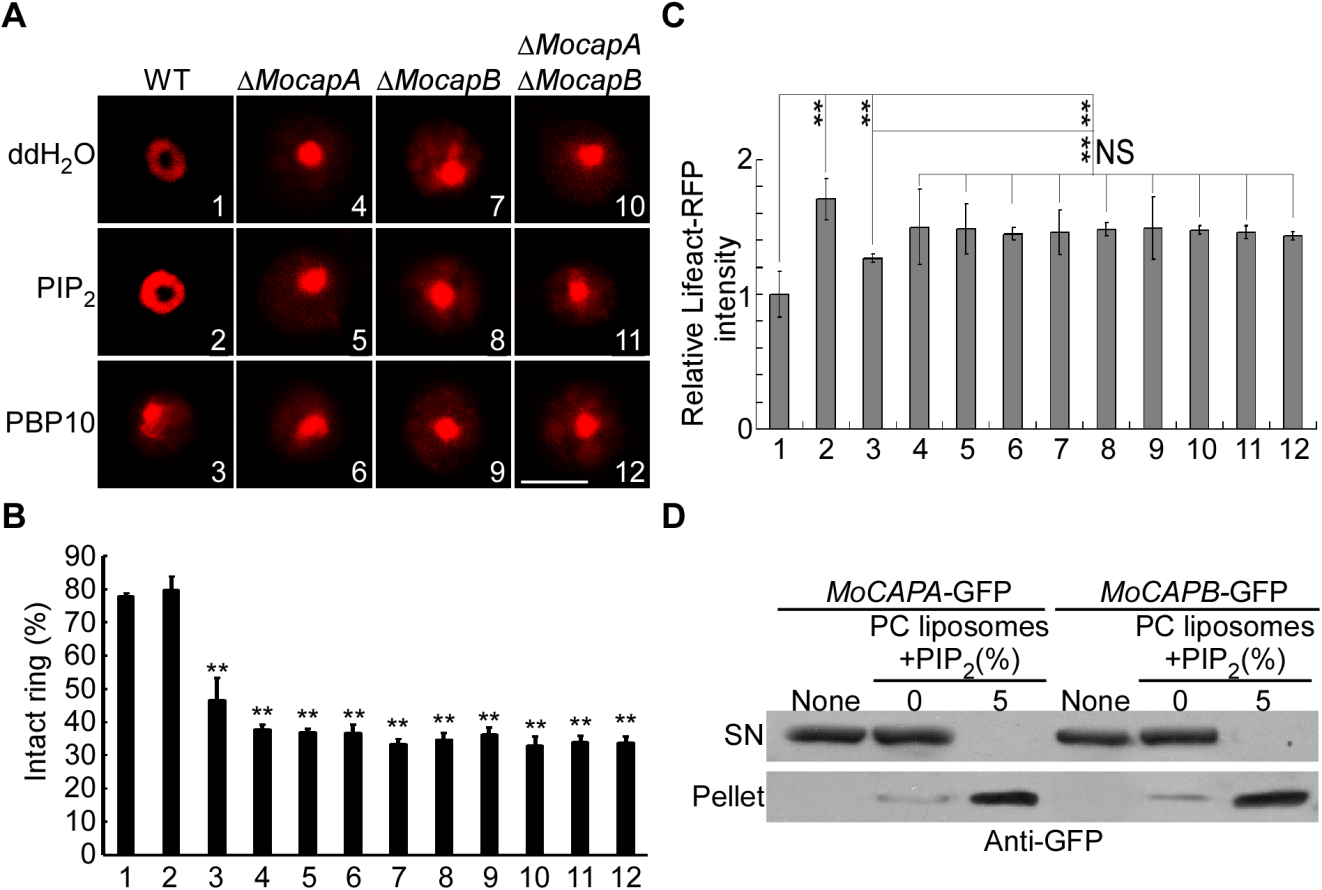
MoCAP proteins regulate the actin organization in response to PIP_2_. (A) Actin filament morphology in appressoria treated with PIP_2_ and PBP10. Transformants of WT, Δ*MocapA*, Δ*MocapB*, and Δ*MocapA*Δ*MocapB* expressing Lifeact-RFP were treated with PIP_2_ and PBP10. Treat with water as a control. (B) Bar chart to show percentage of appressoria containing intact F-actin rings after 12 h. The experiment was repeated three times, (**, *p*<0.01), n = 100. (C) Quantitative analysis of lifeact-RFP was made by using Image J software (rsbweb.nih.gov/ij). For all images, the threshold was auto set (*Analyze-Threshold-Auto*) and the base area of the appressoria surrounding the appressorium pore was drawn inside the appressoria (*Analyze-Measure*). For each strain, at least 10 appressoria were analyzed. The experiments were repeated three times showing similar results. Error bars represent the SD and double asterisks indicate statistically significant differences (**, *p*<0.01). (D) PIP_2_-MoCAP binding assay. Total proteins extracted from strains expressing *MoCAPA*-GFP or *MoCAPB*-GFP was tested in the presence of lipid control PC liposomes or PC liposomes plus PIP_2_. The supernatant (SN) and pellet were both detected by an anti-GFP antibody. Bar = 5 μm.

Many eukaryotic actin-binding proteins are regulated by phosphatidylinositol phosphates (PIP) [8], such as phosphatidylinositol 4,5-bisphosphate (PIP_2_), that interacts with CAP proteins of plant and animal cells [29]. We tested whether this is also the case for MoCAP in *M*. *oryzae*. When exogenous PIP_2_ was applied to the wild-type cells, an enhanced actin ring formation was observed (Fig 10A, 10B and 10C). However, no significant differences in actin organization were seen in the Δ*Mocap* mutants. In addition, polyphosphoinositide-binding peptide (PBP10), a PIP_2_ inhibitor, can effectively reduce the cellular PIP_2_ levels by suppressing PIP_2_ binding capability [44–46]. Consistent with other models, PBP10 inhibited the formation of the actin ring (about 54%) in the wild type cell, mimicking the phenotypes of the Δ*Mocap* mutants (Fig 10A, 10B and 10C). Variations of different cellular PIP_2_ levels also had a role in the regulation of the actin cytoskeleton. Higher PIP_2_ levels lead to the enhanced actin ring formation, whereas reduced PIP_2_ levels blocked this processes. In contrast, the Δ*Mocap* mutants were less sensitive to PIP_2_, suggesting that MoCAP could be the direct target of PIP_2_. Further, we demonstrated that both the MoCapA-GFP and MoCapB-GFP fusion proteins could bind to PIP_2_ (Fig 10D). Taken together, these results indicate that MoCAP proteins regulate the actin organization in response to PIP_2_ signaling.

## Discussion

Our previous studies found that the actin-regulating kinase MoArk1 regulates endocytosis and membrane trafficking and is required for conidial development, stress resistance, and pathogenicity in *M*. *oryzae* [36]. The Ark1 and Prk1 serine/threonine kinases function through phosphorylation of a number of endocytic or actin binding proteins, including Pan1p, Sla1p, Scd5p, Yap1801/2p, Ent1/2p, and Bni1p [24, 25, 47, 48]. In this study, we found that MoArk1 interacts and phosphorylates MoCAP proteins that may provide a mechanism for how MoArk1 functions. To our knowledge, it is the first report that CAP proteins are targets of phosphorylation by Ark1.

Deletion of *MoCAPA* and *MoCAPB* resulted in various defects in rice blast fungus, including reduction in colony growth and conidiophore formation, abnormal mycelia and conidia shapes, and retardation of penetration peg formation and infection hyphae growth. There were no significant phenotypic differences between the single and double deletion mutants, which is consistent with previous studies in other model systems [1, 6]. For example, in the budding yeast, deletion of one CAP subunit leads to a loss of functional protein [6, 13]. This may be due to the possibility that a single CAP subunit might be non-functioning without its heterodimeric partner.

The actin ring which is located at the base of appressorium surrounding the appressorium pore facilitates plant infection [33, 35]. In this study, MoCAP proteins directly interact with and regulate MoAct1. In the Δ*Mocap* mutants, regular actin rings could not form in appressorium compared with wild type. The altered actin distribution and decreased actin intensity in the Δ*Mocap* mutants could lead to the reduced penetration ability of appressoria and thus reduced pathogenicity.

The Δ*Mocap* mutants had an altered actin organization, which modulates endocytosis. The distribution of cortical actin patches in hyphae was completely disrupted in the Δ*Mocap* mutants, which could lead to the defect in endocytosis. In addition, since actin cables are important in vesicle trafficking, the less numerous, less intense, and shorter cables seen in the Δ*Mocap* mutants could also result in defect in endocytic transport. Endocytosis are involved in transporting and scavenging membrane proteins and lipids, absorption of nutrients, regulation of membrane receptors and maintenance of cell polarity in eukaryotic cells [49, 50]. The Δ*Mocap* mutants had an altered endocytosis, which also could be the cause of defects in mycelia polar growth, conidiophore formation, conidium morphology and infection hyphae growth. This is similar to the corn (*Zea mays* L.) smut fungus *Ustilago maydis* in which endocytosis is essential for recognition of the partner at the beginning of the pathogenic program, and for spore formation, germination, and pathogenesis on maize [51].

In this study, F-actin capping proteins MoCapA and MoCapB interact with and are phosphorylated by MoArk1. We also found that MoCapA and MoCapB regulation of actin cytoskeleton and endocytosis was governed by MoArk1-mediated phosphorylation. The constitutively phosphorylated and dephosphorylated MoCapA and MoCapB were all impaired in functions for actin cytoskeleton, endocytosis, growth sporulation capacity and pathogenicity. This demonstrates that MoCAP proteins phosphorylation mediated by MoArk1 plays an important role in protein function. All of the phosphorylated and dephosphorylated states are required for normal functions of MoCapA and MoCapB. In addition, we found the dephosphorylated MoCAP proteins could also function to partially rescue the defects of Δ*MocapA* and Δ*MocapB*, and to partially suppress the defects of Δ*Moark1*. Interestingly, constitutively phosphorylated MoCAP proteins appeared to exacerbate Δ*Moark1* defects. The results suggest that MoCapA and MoCapB are down-stream targets of MoArk1 and whose functions are negatively regulated by MoArk1 through protein phosphorylation. This is similar to *S*. *cerevisiae* Ark1p and Prk1p substrate regulation mechanisms, including Pan1p, Sla1p, Ent1p and Ent2p in *S*. *cerevisiae* [25–27, 52]. These proteins are assembled into the endocytic coat with actin. Ark1p and Prk1p disrupt the activities of these proteins by uncoupling them from the endocytic coat at the late stage of endocytosis. In addition, the phosphorylation by Prk1 inhibits the ability of Pan1 to activate the Arp2/3 complex, thus shutting off Arp2/3-mediated actin polymerization on endocytic vesicles [53]. Moreover, consistent with our studies, the constitutively dephosphorylated state of Ent1p could suppress defects of the Ark1p/Prk1p kinases double mutant *ark1*Δ*prk1*Δ in growth, endocytosis and actin localization, and the constitutively phosphorylated state could exacerbate the defects seen in the *ark1*Δ*prk1*Δ [25].

**Fig 11:**
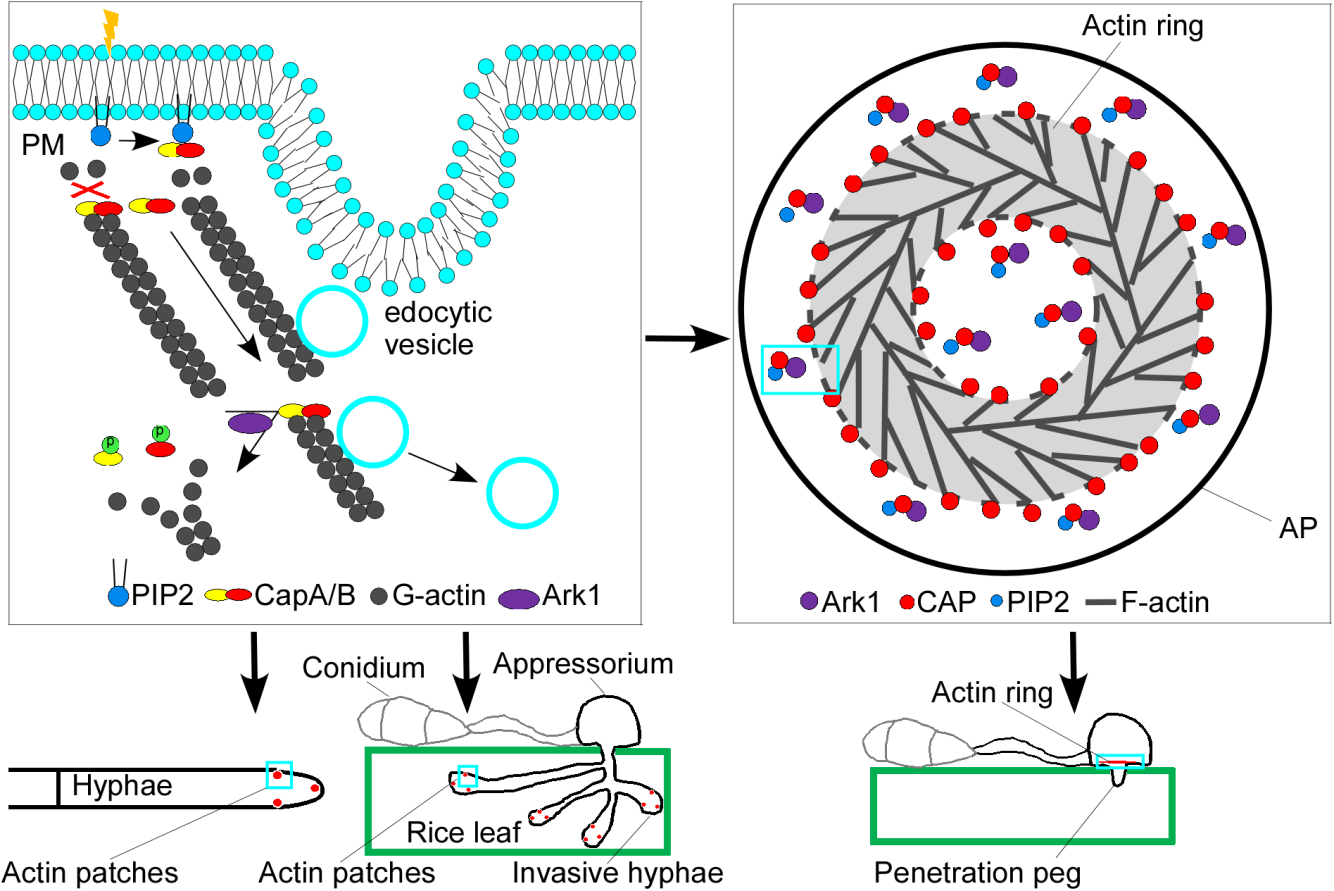
A proposed model for the functions of MoCAP proteins. The schematic diagram for interactions and functions of MoCAP, MoArk1, and PIP_2_ in endocytosis, actin dynamics, growth, and pathogenicity. Unphosphorylated MoCapA and MoCapB bind to the actin filament to block addition and loss of the actin subunits. Upon binding by PIP_2_, MoCAP proteins are detached from actin filaments, and the actin polymerization is enhanced by additions of more actin subunits. After being pinched off the membrane, the endocytic vesicle moves along the actin cable. MoCAP phosphorylation mediated by MoArk1 causes dissociation of MoCAP proteins from actin filaments, facilitating the dissociation of the actin cable from the endocytic vesicle, making it possible for the vesicle to fuse to the endosome.

PIP_2_ serves as a lipid anchor that attaches the cytoskeleton to the plasma membrane and shapes processes (e.g., endo- and exocytosis) that require membrane reorganization [54]. PIP_2_ stabilizes or activates many membrane proteins, such as ion channels and transporters [55], and could also serve as a second messenger to bind CAP proteins, which inhibits their capping activities [28]. Our study supported this conclusion by demonstrating that PIP_2_ modulates the actin organization via interactions with MoCapA and MoCapB proteins. The addition of exogenous PIP_2_ resulted in formation of an enhanced actin ring, whereas the addition of PIP_2_ inhibitor PBP10 mimicked the actin phenotype observed in Δ*Mocap* mutants. Thus, during appressorium formation, MoCAP may likely block actin and toroidal F-actin network assembly in the center of the appressorium, which is regulated by PIP_2_. This has a very important implication in appressoria formation and host penetration by *M*. *oryaze* [33, 56]. Collectively, MoCAP proteins, whose functions are regulated by actin-regulating kinase MoArk1 and PIP_2_, are involved in endocytosis and the maintenance of actin dynamics that directly or indirectly govern fungal growth, conidiation and pathogenicity (Fig 11).

The combined evidence support that CAP proteins and their regulation by Ark1, including MoCAP and MoArk1, and PIP_2_ play important roles in growth, differentiation, and pathogenicity. The regulatory relationship between MoCAP proteins and MoArk1 discovered by this study is a novel finding, but the phosphorylated sites of MoCAP proteins are relatively conserved in other fungal CAP proteins orthologs (S12 Fig). However, whether this regulatory relationship between Ark1 and CAP orthologs can be expanded in other fungi still remain to be investigated, after all, each fungus has its specificity. In addition, several other molecules also influence CAP protein functions, either by binding directly to CAP proteins or by binding to filament-barbed ends [5, 9, 32]. Moreover a diverse and unrelated group of proteins also interact with CAP proteins through the conserved CAP interaction motif to affect their functions. These proteins, including Arp2/3 and myosin I linker [57], CD2‑associated protein [58] and the WASH (WASP and SCAR homologue) complex subunit FAM21 [59], all recruit CAP proteins to specific subcellular locations and modulate their actin-capping activities. Whether these CAP-regulating protein paralogs are present in *M*. *oryzae* and if they have any functions in appressorial actin ring formation or fungal pathogenicity, remain to be investigated. Nevertheless, our studies of MoCAP and MoArk1 and their functions may reveal novel strategies for rice blast management.

## Materials and methods

### Strains and culture conditions

*M*. *oryzae* Guy11 strains were used as the wild type strains for transformation and all strains were maintained on complete medium (CM) plates at 28°C. For conidiation, mycelium blocks were maintained on SDC (100 g of straw, 40 g of corn powder, 15 g of agar in 1 L of distilled water) agar media at 28°C for 7 days in the dark followed by 3 days continuous illumination under fluorescent light [60].

### Gene disruption and complementation

*MoCAPA* and *MoCAPB* targeted gene replacement was performed using the split marker method [58]. For *MoCAPB* disruption, primers HYG-F/HY-R and YG-F/HYG-R were used to amplify the split *hph* template from the vector pKOV21 [61]. Primers HYG-F and HYG-R contain flanking sequence adaptors. The 1.5 kb flanking sequences of *MoCAPA* and *MoCAPB* genes were retrieved from the *M*. *oryzae* genome database (www.broad.mit.edu/annotation/fungi/magnaporthe) at the Broad Institute and were amplified by using specific primers with adaptor sequences (S4 Table). Protoplasts were transformed with *MoCAPB* deletion cassette. For *MoCAPA* disruption, a similar method was used, except that the split template was replaced by neomycin (*NEO*). Double disruption of *MoCAPA* and *MoCAPB* was performed by transforming the *MoCAPA* deletion cassette into the *MoCAPB* deletion mutant. For selecting hygromycin- or neomycin-resistant transformants, CM plates were supplemented with 250 μg/ml hygromycin B (Roche, USA) or neomycin 400 μg/ml (Ameresco, USA).

For complementation assays, the *MoCAPA* and *MoCAPB* gene containing 1.5 kb native promoter were amplified and cloned into pYF11. The resulting constructs pYF11-*MoCAPA* and pYF11-*MoCAPB* were introduced by transformation into the Δ*MocapA* and Δ*MocapB* mutants, respectively. Bleomycin-resistant transformants were isolated and verified by PCR.

### Assays for vegetative growth, conidiation, appressorium formation

Small agar blocks were cut from the edge of 4-day-old cultures and placed onto fresh media (CM, MM, OM and SDC) [39] for culturing in the dark at 28°C.

For conidia production, mycelia were grown in the dark on SDC medium at 28°C for 7 days, followed by constant illumination for 3 days. Conidia were harvested from 10-day-old cultures, filtered through three layers of lens paper and resuspended to a concentration of 5 x 10^4^ spores per milliliter in sterile water. For appressorium formation, droplets (30 μl) of conidial suspension were placed on hydrophobic cover slips and incubated at room temperature as described previously [62]. Appressorium formation was examined after incubation for 24 h.

### Virulence test and infection process observation

To investigate the infection process, conidial suspension with a concentration of 5 x 10^4^ conidia/ml in a 0.2% (w/v) gelatin solution were spotted on lower epidermis of barley leaves, and then incubated in a moist and dark chamber at 28°C. After inoculation, microscopy observations were performed at 24 h after inoculation, respectively. Two-week-old rice seedlings (*oryzae sativa* cv. CO-39) were sprayed with conidial suspension of 5 x 10^4^ conidia/ml and incubated as described previously [61]. Lesion formation was examined at 7 days after inoculation.

### Staining

FM4-64 and rhodamine-conjugated phalloidin staining was conducted following procedures previously described [63, 64].

Calcofluor White (CFW) staining was performed by using fluorescent brightener 28 (10 μg/ml, Sigma-Aldrich) for the microscopy of conidia. The conidia were washed down from the SDC plate and stained with 10 μg/ml CFW and viewed under the fluorescence microscope.

### Phospholipid binding assay

The interaction of MoCAP with phospholipids PIP_2_ was tested by protein–lipid assay according to the methods described by Mustafa et al [65]. Briefly, 1 μg protein, 20 μl 1 mM PolyPIPosomes containing 5% PIP_2_ (Echelon Biosciences), and 1 ml binding buffer (50 mM Tris, pH 7.5, 150 mM NaCl, 0.05% Nonidet P-40) were mixed with rotation for 1 h at room temperature. The mixture was precipitated at 13,000 rpm for 10 min and the liposome pellet was recovered and re-suspended in 1 ml of binding buffer. The step was repeated for three times and the final pellet was resuspended by 20 μl binding buffer. Both the pellet and the condensed supernatant were subjected to SDS-PAGE electrophoresis and Western blot analysis with an anti-GFP antibody (1:5,000; Abmart, China). Proteins mixed with and without PolyPIPosomes were used for controls.

### Co-immunoprecipitation

To confirm the interactions of MoCapA, MoCapB, and MoAct1 *in vivo*, the cDNA of *MoACT1* was cloned into pKNFLAG, the cDNA of *MoCAPA* was cloned into pKNFLAG or pKNRG, and the cDNA of *MoCAPB* was cloned into pKNRG. The *MoCAPA*-3xFLAG and *MoCAPB*-GFP fusion constructs were introduced by co-transformation into protoplasts of the wild-type strain. Total proteins were isolated from transformants expressing both *MoCAPA*-3xFLAG and *MoCAPB*-GFP and incubated with anti-Flag M2 affinity resins (Sigma Aldrich). Proteins bound to M2 resins were eluted after a series of washing steps as manufacture’s instruction. Western blots of total proteins and elution from M2 resins were detected with anti-FLAG (Sigma Aldrich) and anti-GFP (Abmart) antibodies using the ECL Supersignal system (Pierce, Rockford, IL). Similar methods were used to detect interactions of *MoACT1*-3xFLAG/ *MoCAPA*-GFP and *MoACT1*-3xFLAG/ *MoCAPB*-GFP.

### Yeast two-hybrid assays

The bait constructs were generated by cloning *MoARK1* full-length cDNAs into pGBKT7. The *MoCAPA* and *MoCAPB* cDNA were cloned into pGADT7 as the prey constructs, respectively (see primers in S4 Table). The resulting prey and bait constructs were confirmed by sequencing analysis and introduced in pairs into yeast strain AH109 as the description of BD library construction & screening kit (Clontech, USA). The Trp+ and Leu+ transformants were isolated and assayed for growth on SD-Trp-Leu-His-Ade medium. Yeast strains for positive and negative controls were from the two-hybrid assay kit.

For bait constructs, *MoACT1* and *MoCAPB* cDNA was cloned into pGADT7. The prey constructs of *MoACT1* and *MoCAPA* were generated by cloning their cDNA into pGBKT7. The resulting prey and bait constructs were introduced in pairs (BD- *MoACT1*/AD-*MoCAPB*, AD-*MoACT1*/BD-*MoCAPA*, AD-*MoCAPB* /BD-*MoCAPA*) into yeast strain AH109. The Trp+ and Leu+ transformants were isolated and assayed for growth on SD-Trp-Leu-His medium and the expression of the LacZ reporter gene was detected according to the instruction provided by the manufacturer (Stratagene).

### BiFC assays for the MoCAP-MoArk1 interaction

The *MoCAPA*-YFP^N^ and *MoCAPB*-YFP^N^ were generated by cloning the *MoCAPA* and *MoCAPB* fragments into pHZ65. *MoARK1* was cloned into pHZ68 to generate the *MoARK1*-YFP^C^ fusion construct. Plasmids *MoCAPA*-YFP^N^ and *MoARK1*-YFP^C^ were introduced by co-transformation into protoplasts of Guy11. Plasmids *MoCAPB*-YFP^N^ and *MoARK1*-YFP^C^ were also introduced into protoplasts of Guy11. Transformants resistant to both hygromycin and zeocin were isolated and characterized.

### *In vitro* GST pull-down assays

To construct GST-fusion plasmids, *MoARK1* were inserted into the vector pGEX4T-2 (GE Healthcare Life Science). To construct His-fusion plasmid, *MoCAPA* and *MoCAPB* were inserted into the vector pET-32a (Navogen), respectively. Pull-down assay was carried out using profound pull-down GST protein-protein interaction kit (Pierce) according to the manufacturer’s instructions. Briefly, GST or GST-*MoARK1* was expressed in *E*. *coli* strain BL21 (DE3). Soluble proteins were incubated with 50 μl glutathione agarose beads (Invitrogen) for 2 h at 4˚C. The beads were washed five times and then incubated with an equal amount of bacterial lysates containing His-MoCapA or His-MoCapB for another hour at 4˚C. The beads were washed five times again, and the presence of His-MoCapA or His-MoCapB was detected by immuno-blotting using anti-His antibody (Abmart).

### Exogenous PIP_2_ and PBP10 treatment

Conidia were treated with 1 mM PolyPIPosomes containing 5% PIP_2_ (Echelon Biosciences, Germany) or 1 μM PBP10 (Echelon Biosciences, Germany) and treated spores were inoculated on the hydrophobic glasses and observed under the fluorescence microscope after 12 hours.

### Generation of the *MoCAPA*^ΔA1M^-GFP, *MoCAPA*^ΔA2M^-GFP, *MoCAPB*^ΔBM^-GFP *MoCAPA*^S85A^-GFP, *MoCAPA*^S85D^-GFP, *MoCAPB*^S285A^-GFP, *MoCAPB*^285D^-GFP and H1-RFP constructs

To generate the *MoCAPA*^ΔA1M^-GFP construct, PCR products containing the native promoter of *MoCAPA* were amplified with primers MGG_12818A1F/ MGG_12818A1R (S4 Table), and introduced by co-transformation with fragments amplified with primers MGG_12818B1F/ MGG_12818B1R (S4 Table) into the yeast strain XK125 with *Xho*I digested vector pYF11 that contains the bleomycin-resistant gene [66]. Plasmid pYF11-*MoCAPA*^ΔA1M^ was rescued from the resulting Trp+ yeast transformants. The same strategy was used to generate the pYF11- *MoCAPA*^ΔA2M^ vector (PCR products amplified with primers MGG_12818A2F/ MGG_12818A2R and MGG_12818B2F/MGG_12818B2R respectively) and the pYF11-*MoCAPB*^ΔBM^ vector (PCR products amplified with primers MGG_09902AF/ MGG_09902AR and MGG_09902BF/ MGG_09902BR, respectively). The same strategy also was used to generate the continuously phosphorylated constructs (pYF11- *MoCAPA*^S85D^ and pYF11-*MoCAPB*^S285D^) and continuously dephosphorylated constructs (pYF11- *MoCAPA*^S85A^ and pYF11-*MoCAPB*^S285A^).

To generate the P_RP27_-H1-RFP fusion construct, a histone H1 fragment (amplified by H1RfpF and H1RfpR) and a RFP fragment (amplified by RFPh1F and RFPh1R) were introduced by co-transformation with *Xho*I-digested pYF11 into yeast strain XK125 [66]. Plasmid pYF11-H1-RFP was rescued from the resulting Trp+ yeast transformants.

### Phos-tag analysis

The *MoCAPA-GFP* and *MoCAPB-GFP* fusion constructs were transferred respectively into the wild-type strain and Δ*Moark1* mutant. The positive transformants were cultured in liquid CM for 48 h. For protein isolation, about 150 to 200 mg of mycelia were ground into powder in liquid nitrogen and resuspended in 1 ml of extraction buffer (10 mM Tris-HCl [pH 7.5], 150 mM NaCl, 0.5 mM EDTA, 0.5% NP40) to which 1mM PMSF, 10 μl of protease inhibitor cocktail (Sigma), and 10 μl of phosphatase inhibitor cocktail 3 (Sigma) had to be freshly added. For the preparation of the phosphatase-treated Cell lysates, the phosphatase inhibitor cocktail was omitted for 2.5 U/ml alkaline phosphatase (final concentration; P6774; Sigma) and the sample was incubated for 1 h with the addition of 1 mM MgCl_2_ (37°C). Then the samples were resolved on 8% SDS-polyacrylamide gels prepared with 50 μM acrylamide-pendant Phos-tag ligand and 100 μM MnCl_2_ according to the instructions provided by the Phos-tag Consortium. Gels were electrophoresed at 80 V/gel for 3–6 h. Prior to transfer, gels were first equilibrated in transfer buffer containing 5 mM EDTA for 20 min two times and then in transfer buffer without EDTA for 10 min. Protein transfer from the Mn^2+^-phos-tag^TM^ acrylamide gel to the PVDF membrane was performed overnight at 80 V at 4°C, and then the membrane was analyzed by Western blotting (using anti-GFP antibodies).

### MoCapA-GFP and MoCapB-GFP protein purification

The MoCapA-GFP and MoCapB-GFP protein were extracted as previously described in Phos-tag analysis. GFP-Trap A beads were equilibrated in 500 μl dilution buffer (10 mM Tris-HCl [pH 7.5], 150 mM NaCl, 0.5 mM EDTA) with freshly added 1mM PMSF and 5 μl of protease inhibitor cocktail (Sigma). 50 μl bead slurry was resuspended in 500 μl ice cold dilution buffer and spin down at 2500g for 2 minutes at 4°C. The supernatant was discarded and beads washed two more times with 500 μl ice cold dilution buffer. The cell lysate was then added to equilibrated GFP-Trap_A beads and incubated under constant mixing for 4 h at 4°C. The mixture was precipitated at 2500g for 2 minutes at 4°C, washed six times with 500ul washing buffer (10 mM Tris-HCl [pH 7.5], 150 mM NaCl, 0.5 mM EDTA, 1mM PMSF, 5 μl of protease inhibitor cocktail (Sigma), and 5 μl of phosphatase inhibitor cocktail 3 (Sigma)). Following the last wash step, beads were transferred to a new tube and bound proteins were eluted by adding 50 μl 0.4 M glycine pH 2.5 (incubation time: 30 sec under constant mixing) followed by centrifugation. The supernatant was again transferred to a fresh tube and 5 μl 2M Tris base (pH 10.4) was added for neutralization. The supernatant was resuspended in 12.5 μl 5×SDS-Sample buffer, boiled for 10 minutes at 95°C, and analyzed by 10% SDS-PAGE. The gel bands corresponding to the targeted protein were excised from the gel.

### Mass spectrometric analysis

To identify phosphorylated sites of targeted proteins, samples were separated on 10% SDS PAGE. The gel bands corresponding to the targeted protein were excised from the gel, reduced with 10 mM of DTT and alkylated with 55 mM iodoacetamide. Then in gel digestion was carried out with the trypsin/lys-c mix (Promega, USA) in 50 mM ammonium bicarbonate at 37°C overnight. The peptides were extracted using ultrasonic processing with 50% acetonitrile aqueous solution for 5 min and with 100% acetonitrile for 5 min. The extractions were then centrifuged in a speedvac to reduce the volume. A liquid chromatography–mass spectrometry (LC–MS) system consisting of a Dionex Ultimate 3000 nano-LC system (nano UHPLC, Sunnyvale, CA, USA), connected to a linear quadrupole ion trap Orbitrap (LTQ Orbitrap XL) mass spectrometer (ThermoElectron, Bremen, Germany), and equipped with a nanoelectrospray ion source was used for our analysis. For LC separation, an Acclaim PepMap 100 column (C18, 3 μm, 100 Å) (Dionex, Sunnyvale, CA, USA) capillary with a 15 cm bed length was used with a flow rate of 300 nL/min. Two solvents, A (0.1% formic acid) and B (aqueous 90% acetonitrile in 0.1% formic acid), were used to elute the peptides from the nanocolumn. The gradient went from 5% to 40% B in 80 min and from 40% to 95% B in 5 min, with a total run time of 120 min. The mass spectrometer was operated in the data-dependent mode so as to automatically switch between Orbitrap-MS and LTQ-MS/MS acquisition. Survey full scan MS spectra (from m/z 350 to 1800) were acquired in the Orbitrap with a resolution r = 60,000 at m/z 400, allowing the sequential isolation of the top ten ions, depending on signal intensity. The fragmentation on the linear ion trap used collision-induced dissociation at a collision energy of 35 V. Protein identification and database construction were processed using Proteome Discoverer software (1.2version, Thermo Fisher Scientific, Waltham, MA, USA) with the model of SEQUEST. The protein database was self-defined according to the MoCapA amino acid sequence (MTKSTEGRCDFLVASRAVSYQDTFAFGVAPHSVAYSDNADAPFFHNSYPRLAIAPVKTTSQTSSLLRSRRIDIKSISSGDAKVVSKLAPAFERYNEEQFTTVKLPGGSQKVIVSAHNSLGDGRYYDVESSSSFAFDHTTQKASAVQSYALESAHSDLVNGRWRSLYTLDPASGAIDGSIKVDVHYYEDGNVRLLTDKATTATVPSATGSAIVKEIGSSEKKYQEELNRGFTDLSEGAFKGLRRQLPVTRQKIEWDKVASYRLGQDIGGGRRQG) and the MoCapB amino acid sequence (MAADPFDSALDLLRRLDPKHTTRHLNGLMTIVPDLTEDLLSSVDQPLTVRRCKQTGREYLLCDYNRDGDSYRSPWSNEFDPPLDDGPGGLGGVGPQGGNEGAGELGVPGERVRKMEVKANEAFDVYRDLYYEGGVSSVYLWNLDDGFAGVVLLKKAAPQGGNNEGVWDSIHVFEASERGRSTTYRLTSTVILTLSAGGGDSALGDMNLSGNMTRQLEQDMRTAEGDESHIANLGRLVEDMELKMRNLLQEVYFGKAKDVVGDLRSLGSLSEGQRDRDAQREIIGSMQR). MS/MS-based peptide identifications were accepted if they could be established at greater than 95.0% probability, as specified by the Peptide Prophet algorithm.

## Supporting information

S1 Fig The *MoARK1*-3×FLAG transformant, phylogenetic tree analysis and structure prediction of Caps from selected fungi. (A) Western blots of total proteins and proteins eluted from anti-FLAG M2 beads from transformant expressing the *MoARK1*-3×FLAG construct were detected with an anti-FLAG antibody. (B and D) Sequence alignments were performed using the Clustal_W program and the calculated phylogenetic tree was viewed using Mega5.0 Beta program. Neighbor-joining tree with 500 bootstrap replicates of phylogenetic relationships between CapA and CapB homologues in fungi. All of the CapA and CapB proteins were downloaded from the NCBI database and their accession numbers are as following: *M*. *oryzae* (*Magnaporthe oryzae* XP_003717048.1 and XP_003709997.1), *F*. *oxysporum* (*Fusarium oxysporum* EWY88482.1 and EMT62332.1), *G*. *graminis* (*Gaeumannomyces graminis* XP_009216882.1 and XP_009222432.1), *N*. *crassa* (*Neurospora crassa* XP_957550.2 and XP_963750.2), *P*. *fici* (*Pestalotiopsis fici* XP_007836868.1 and XP_007827841.1), *T*. *hemipterigena* (*Torrubiella hemipterigena* CEJ91114.1 and CEJ81715.1), *V*. *dahliae* (*Verticillium dahliae* XP_009648755.1 and XP_009649241.1), *C*. *graminicola* (*Colletotrichum graminicola* EFQ30234.1 and EFQ26548.1), *A*. *niger* (*Aspergillus niger* XP_001394532.2 and EHA26453.1) and *S*. *cerevisiae* (*Saccharomyces cerevisiae* NP_012918.1 and EDV09521.1). (C and E) Prediction of motifs in MoCAP using the Motif Scan website.
(TIF)

S2 Fig The phase specific expression of *MoCAP*. The expression of *MoCAP* was measured by quantitative real-time RT-PCR with cDNA from samplings for infectious growth, vegetative growth, and conidia. The relative abundance of *MoCAP* transcripts during infectious growth (from ungerminated conidia to in planta fungal cells 96 hpi) was normalized by comparing with vegetative growth in liquid CM (Relative transcript level = 1). Each sample was harvested from 10 plants and three independent experiments, each with three replicates, were performed. Significant differences are presented in the figure (*p*< 0.01), and the error bar represents the standard deviation.
(TIF)

S3 Fig MoCAP proteins interact with MoArk1. (A and B) Yeast two-hybrid assays for the interaction between MoCAP proteins and MoArk1. Yeast transformants expressing bait (pGBKT7) and prey (pGADT7) constructs were assayed for growth on SD-His-Leu-Trp plates added with 1 mM 3AT (3-amino-1,2,4-triazole) and SD-Ade-His-Leu-Trp plates and β-galactosidase (LacZ) activities with positive and negative control. (C) Coomassie brilliant blue stained gels of pull-down assays of MoArk1 and MoCAP proteins. MoArk1-GST- or GST-bound glutathione resins were incubated with cell lysates containing MoCapA-His and MoCapB-His, respectively. The precipitation of MoCAP-His, MoArk1-GST and GST were examined by Coomassie brilliant blue staining analysis before incubation (Input) and after wash (Pull-down).
(TIF)

S4 Fig Targeted gene replacement of *MoCAPA and MoCAPB*. (A) Schematic diagram of deletion strategy of *MoCAPA* and *MoCAPB*. *NE* and *EO* indicate parts of the neomycin phosphotransferase gene cassate. *HY and YG* indicate parts of the hygromycin B phosphotransferase gene cassette. *Bgl*II and *Hin*dIII are used for genomic DNAs digestion. The split *HYG* templates for *MoCAPB* disruption and *NEO* templates for *MoCAPA* disruption were amplified and ligated to the specific flanking sequences by overlap-PCR. Protoplasts of the *M*. *oryzae* strain were transformed with each deletion cassette. For double gene replacement, the *MoCAPA* deletion cassette was transformed into the Δ*MocapB* mutant and selected. (B) Southern blot confirmation of the single and double deletion mutants of *MoCAPA* and *MoCAPB*. *Bgl*II-digested genomic DNAs of *MoCAPA* deletion mutants were hybridized with a 1.5 kb 3’-flanking fragment of *MoCAPA*. *Hin*dIII-digested genomic DNAs of *MoCAPB* deletion mutants were hybridized with a 1.5 kb 5’-flanking fragment of *MoCAPB*. *Bgl*II+*Hin*dIII-digested genomic DNAs of the double mutants were hybridized with both probes used in single deletion mutants. WT, the wild-type strain; *MoCAPA*KO1 to *MoCAPA*KO3, the *MoCAPA* deletion mutants; *MoCAPB*KO1 to *MoCAPB*KO3, the *MoCAPB* deletion mutants; DKO1 and DKO2, the *MoCAPA* and *MoCAPB* double deletion mutants. When probed with a 1.5-kb downstream fragment, the putative *MoCAPA* null mutant contained one hybridizing 8.1-kb *Bgl*II-fragment in contrast to the one hybridizing 6.1-kb fragment in the wild-type. Similarly, when probed with a 1.5-kb upstream fragment, the putative *MoCAPA* null mutant contained one hybridizing 6.9-kb *Hin*dIII-fragment in contrast to the one hybridizing 7.6-kb fragment in the wild-type. Both probes used for the single replacement mutants were used for verification of the double gene replacement mutant. Two hybridizing fragments, a 6.1-kb *Bgl*II-fragment and a 6.9-kb *Hin*dIII-fragment, were detected in the double mutants, while a 6.1-kb fragment and a 7.6-kb *Hin*dIII-fragment were detected in the wild-type strain.
(TIF)

S5 Fig MoCAP proteins are required for growth. (A) The Δ*Mocap* mutants displayed reduced mycelial growth. The indicated strains were inoculated on complete medium (CM), minimal medium (MM), straw decoction and corn agar (SDC), and oatmeal agar (OM) and cultured at 28°C for 7 days in the dark. (B) The Δ*Mocap* mutants displayed altered colony morphology. The indicated strains on CM media following incubation of plates at 28°C for 7 days in the dark. (C) The Δ*Mocap* mutants formed small compact mycelia masses. The indicated strains incubated in liquid CM for 48 h.
(TIF)

S6 Fig MoCAP proteins are important for conidial morphology. (A-C) Conidia shape comparison. Conidia of the indicated strains were harvested from SDC medium, stained with calcofluor white, and observed by fluorescence microscope. Bar = 10 μm.
(TIF)

S7 Fig The F-actin-capping motifs are essential for the interactions between MoCAP proteins and MoAct1. (A) Schematic representation of motifs deleted in MoCapA and MoCapB. (B, C and D) MoCapA^ΔA1M^, MoCapA^ΔA2M^ and MoCapB^ΔBM^ do not interacts with MoAct1. Yeast transformants expressing bait (pGBKT7) and prey (pGADT7) constructs were assayed for growth on SD-His-Leu-Trp plates added with 1mM 3AT and β-galactosidase (LacZ) activities with positive and negative control. MoCapA^ΔA1M^ (MoCapA^ΔVHYYEDGNV^, MoCapA without F-actin-capping A1 motif), MoCapA^ΔA2M^ (MoCapA^ΔKGLRRQLPVTR^, MoCapA without F-actin-capping A2 motif), MoCapB^ΔBM^ (MoCapB^ΔCDYNRD^, MoCapB without F-actin-capping B motif).
(TIF)

S8 Fig Purification of GFP tagged MoCapA and MoCapB proteins. MoCapA-GFP and MoCapB-GFP protein were purified form *MoCAPA-GFP* and *MoCAPB-GFP* expressing cells. MoCapA-GFP and MoCapB-GFP protein were separated by SDS-PAGE and subject for LC-MS/MS analysis as mentioned in the materials and methods.
(TIF)

S9 Fig Mass spectrometric analysis of MoCapA and MoCapB phosphopeptides. (A and B) Mass spectrometry showed that the serine at the 85th residue (S85) of MoCapA and the serine at the 285th residue (S285) of MoCapB were phosphorylated. An analysis of a phosphopeptide from purified MoCapA-GFP and MoCapB-GFP expressed in the wild type. The product ion spectrum of *m*/*z* 648.8 identifies the peptide as mono-phosphorylated VVSpKLAPAFER and pinpoints serine 85 as the site of phosphorylation of MoCapA. For MoCapA, [M+2H] represented the identified peptide fragment with Pi group (corresponding molecular weight 1296.36). When one amino acid (the 1^st^ one is Arg) was removed from the carboxyl terminal of the peptide fragment during MS analysis, b10^+^ (with 1 positive charge) was produced with a molecular weight of 1122.47. Then, amino acid residues or NH_3_/H_2_O/Pi groups were subsequently removed to produce b_9_^+^, b_10_^+^-NH_3_, b_10_^+^-NH_3_-Pi, and so on. When amino acid residues or NH_3_/H_2_O/Pi were removed from the amino terminal, y series fragments were produced. From the molecular weight y_8_^+^-H_2_O, we can calculated that a phosphorylated serine was presented in the fragment (MW of y_8_ is equal to y_10_ with Val and Ser-Pi residues removed). Also, from the molecular weight of b_3_^+^-NH_3_-P and b_3_^2+^-H_2_O, we can calculated that a phosphorylated serine was presented. The product ion spectrum of *m*/*z* 742.3 identifies the peptide as mono-phosphorylated DAQREIIGSpMQR and pinpoints serine 285 as the site of phosphorylation of MoCapB. By the same calculation process, the phosphorylated serine in MoCapB (MW of b_7_ is equal to b_8_ with Gly and Ser-Pi residues removed) was calculated.
(TIF)

S10 Fig Western blot analysis of MoCapA and MoCapB in phosphorylation site mutants. (A) Western blot analysis of MoCapA in phosphorylation site mutants with anti-GFP. (B) Western blot analysis of MoCapB in phosphorylation site mutants with anti-GFP.
(TIF)

S11 Fig Western blot analysis of constitutively dephosphorylated and phosphorylated MoCapA and MoCapB in Δ*Moark1*. (A) Western blot analysis of constitutively dephosphorylated and phosphorylated MoCapA in Δ*Moark1* with anti-GFP. (B) Western blot analysis of constitutively dephosphorylated and phosphorylated MoCapB in Δ*Moark1* with anti-GFP.
(TIF)

S12 Fig Alignments of CapA and CapB. (A) Sequence alignments of CapA using the Clustal_W program suggested that other CapAs also have the conserved serine like the 85th residue (S85) of MoCapA. All of the CapA proteins were downloaded from the NCBI database and their accession numbers are as following: *M*. *oryzae* (*Magnaporthe oryzae* XP_003717048.1), *S*. *cerevisiae* (*Saccharomyces cerevisiae* NP_012918.1). *C*. *graminicola* (*Colletotrichum graminicola* XP_008094254.1), *F*. *oxysporum* (*Fusarium oxysporum* EWY88482.1), *P*. *fici* (*Pestalotiopsis fici* XP_007836868.1), *P*. *chlamydosporia* (*Pochonia chlamydosporia* XP_018149953.1), *H*. *minnesotensis* (*Hirsutella minnesotensis* KJZ78485.1), *S*. *chartarum* (*Stachybotrys chartarum* KEY66866.1), *P*. *lilacinum* (*Purpureocillium lilacinum* XP_018182328.1), *B*. *bassiana* (*Beauveria bassiana* XP_008598938.1), *M*. *bolleyi* (*Microdochium bolleyi* KXJ88500.1), *M*. *album* (*Metarhizium album* KHO01770.1) and *V*. *alfalfae* (*Verticillium alfalfae* XP_003001819.1) (B) Sequence alignments of CapB using the Clustal_W program suggested that other CapAs also have the conserved serine like the 285th residue (S285) of MoCapB. All of the CapB proteins were downloaded from the NCBI database and their accession numbers are as following: *M*. *oryzae* (*Magnaporthe oryzae* XP_003709997.1), *S*. *cerevisiae* (*Saccharomyces cerevisiae* EDV09521.1). *C*. *graminicola* (*Colletotrichum graminicola* EFQ26548.1), *F*. *oxysporum* (*Fusarium oxysporum* EMT62332.1), *P*. *fici* (*Pestalotiopsis fici* XP_007827841.1), *P*. *chlamydosporia* (*Pochonia chlamydosporia* XP_018140901.1), *H*. *minnesotensis* (*Hirsutella minnesotensis* KJZ76596.1), *S*. *chartarum* (*Stachybotrys chartarum* KEY65176.1), *P*. *lilacinum* (*Purpureocillium lilacinum* XP_018180365.1), *B*. *bassiana* (*Beauveria bassiana* KGQ08932.1), *M*. *bolleyi* (*Microdochium bolleyi* KXJ85966.1), *M*. *acridum* (*Metarhizium acridum* XP_007810482.1) and *V*. *dahliae* (*Verticillium dahliae* XP_009649241.1)
(TIF)

S1 Table Putative MoArk1-interacting proteins identified by affinity purification. (DOCX)

S2 Table Statistical analysis of the growth and conidiation of the phosphorylation site mutants. (DOCX)

S3 Table Comparison of the growth and conidiation among Δ*Moark1* and phosphorylation site mutants. (DOCX)

S4 Table Primers used in this study. (DOC)

S1 Video Actin morphologies in hyphae of WT. (AVI)

S2 Video Actin morphologies in hyphae of Δ*MocapA* mutant. (AVI)

S3 Video Actin morphologies in hyphae of Δ*MocapB* mutant. (AVI)

S4 Video Actin morphologies in hyphae of Δ*MocapA*Δ*MocapB* mutant. Micrographs of F-actin were observed by express Lifeact-RFP in the indicated strains. Hyphae of the indicated strains expressing Lifeact-RFP were cultured in liquid CM for 48 h. Observations were made with a confocal fluorescence microscope (Zeiss LSM710, 63×oil). Bar = 5 μm.
(AVI)
