# Supplementary figures and images for: MoCAP proteins regulated by MoArk1-mediated phosphorylation coordinate endocytosis and actin dynamics to govern development and virulence of Magnaporthe oryzae
Source: PLoS Genet. 2017 May 25;13(5):e1006814. doi: 10.1371/journal.pgen.1006814 (PMC5466339; doi:10.1371/journal.pgen.1006814)

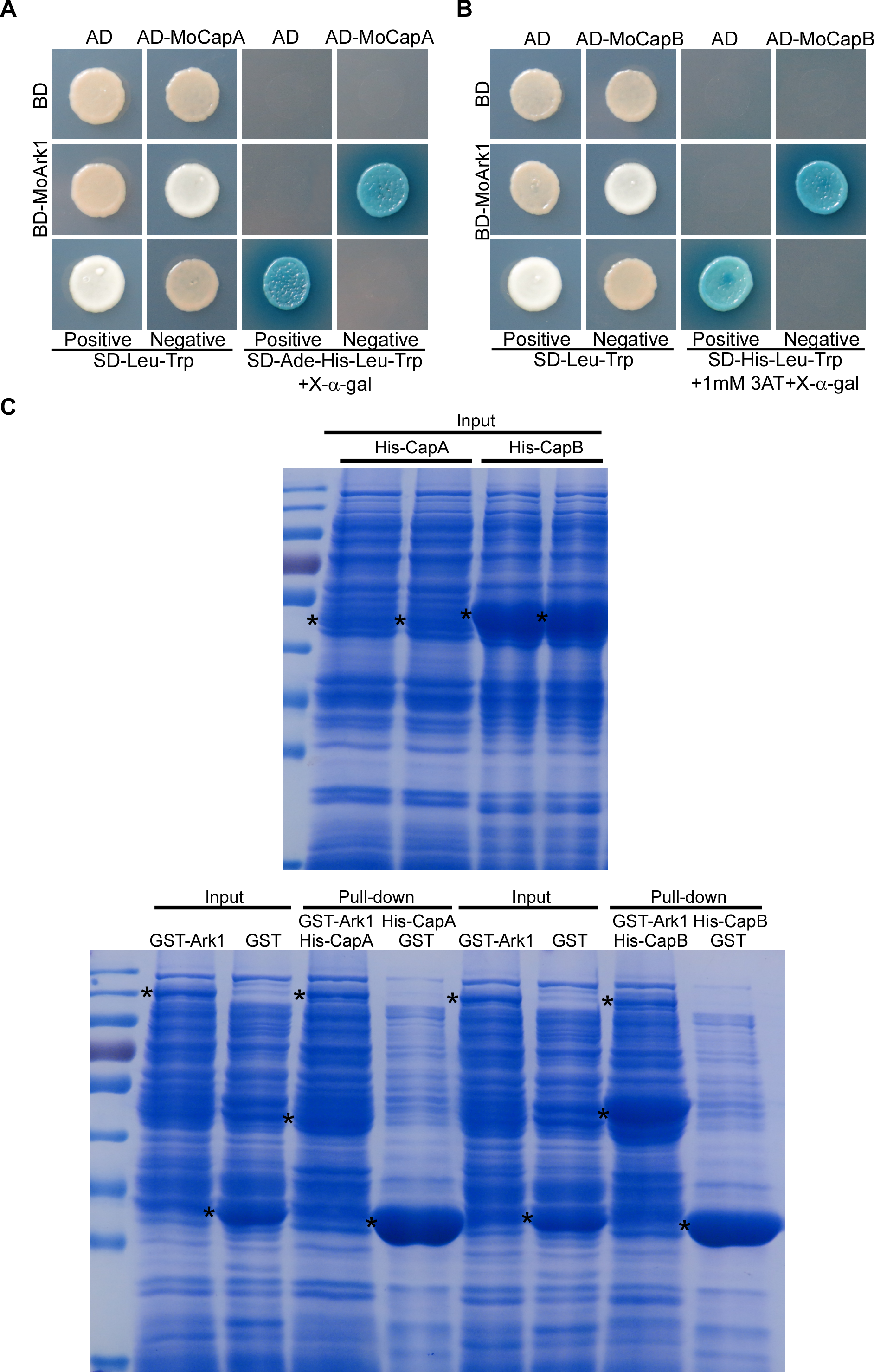

Supplement: S3 Fig — (A and B) Yeast two-hybrid assays for the interaction between MoCAP proteins and MoArk1. Yeast transformants expressing bait (pGBKT7) and prey (pGADT7) constructs were assayed for growth on SD-His-Leu-Trp plates added with 1 mM 3AT (3-amino-1,2,4-triazole) and SD-Ade-His-Leu-Trp plates and β-galactosidase (LacZ) activities with positive and negative control. (C) Coomassie brilliant blue stained gels of pull-down assays of MoArk1 and MoCAP proteins. MoArk1-GST- or GST-bound glutathione resins were incubated with cell lysates containing MoCapA-His and MoCapB-His, respectively. The precipitation of MoCAP-His, MoArk1-GST and GST were examined by Coomassie brilliant blue staining analysis before incubation (Input) and after wash (Pull-down). (TIF) [file pgen.1006814.s003.tif]

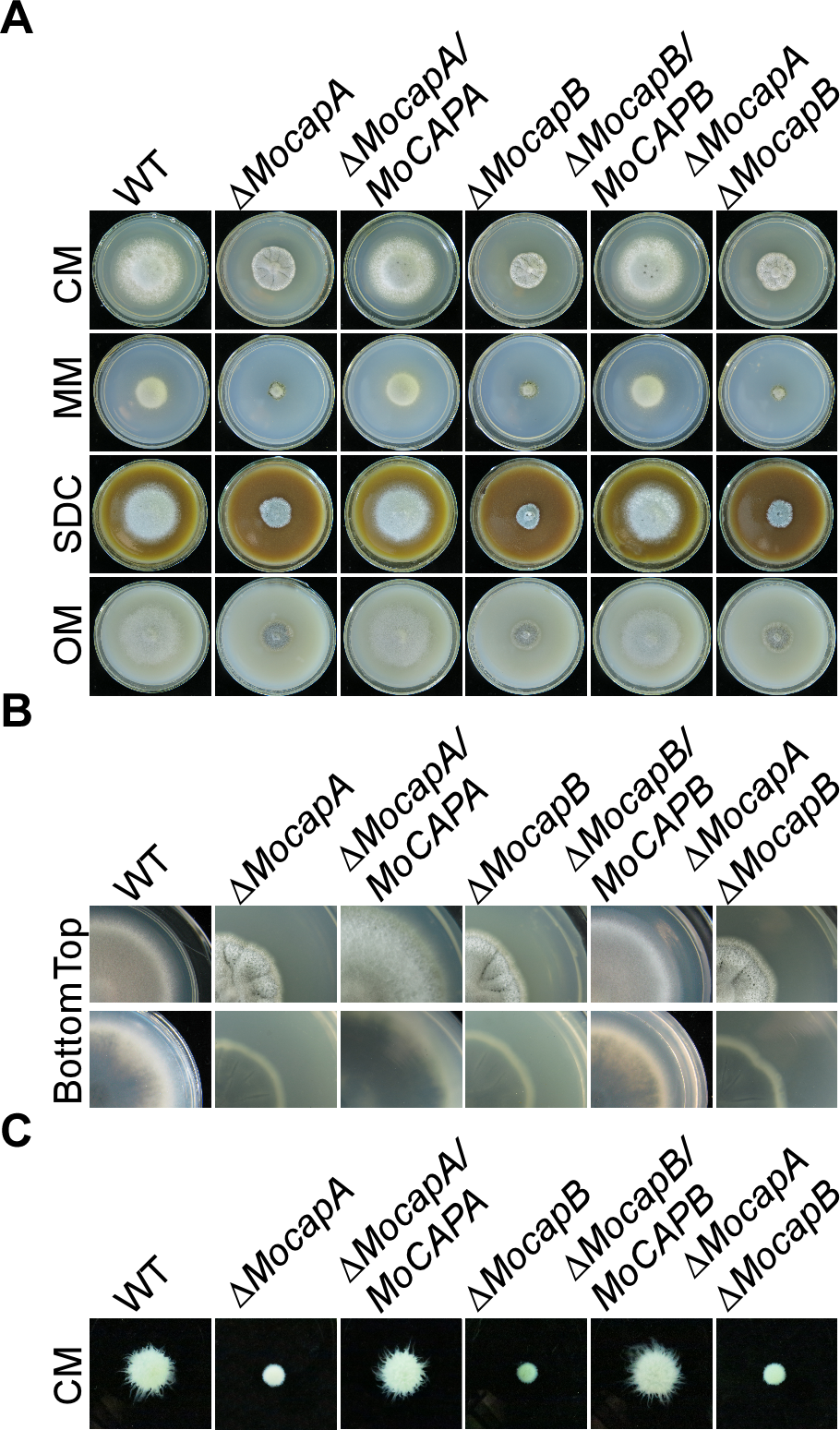

Supplement: S5 Fig — (A) The ΔMocap mutants displayed reduced mycelial growth. The indicated strains were inoculated on complete medium (CM), minimal medium (MM), straw decoction and corn agar (SDC), and oatmeal agar (OM) and cultured at 28°C for 7 days in the dark. (B) The ΔMocap mutants displayed altered colony morphology. The indicated strains on CM media following incubation of plates at 28°C for 7 days in the dark. (C) The ΔMocap mutants formed small compact mycelia masses. The indicated strains incubated in liquid CM for 48 h. (TIF) [file pgen.1006814.s005.tif]

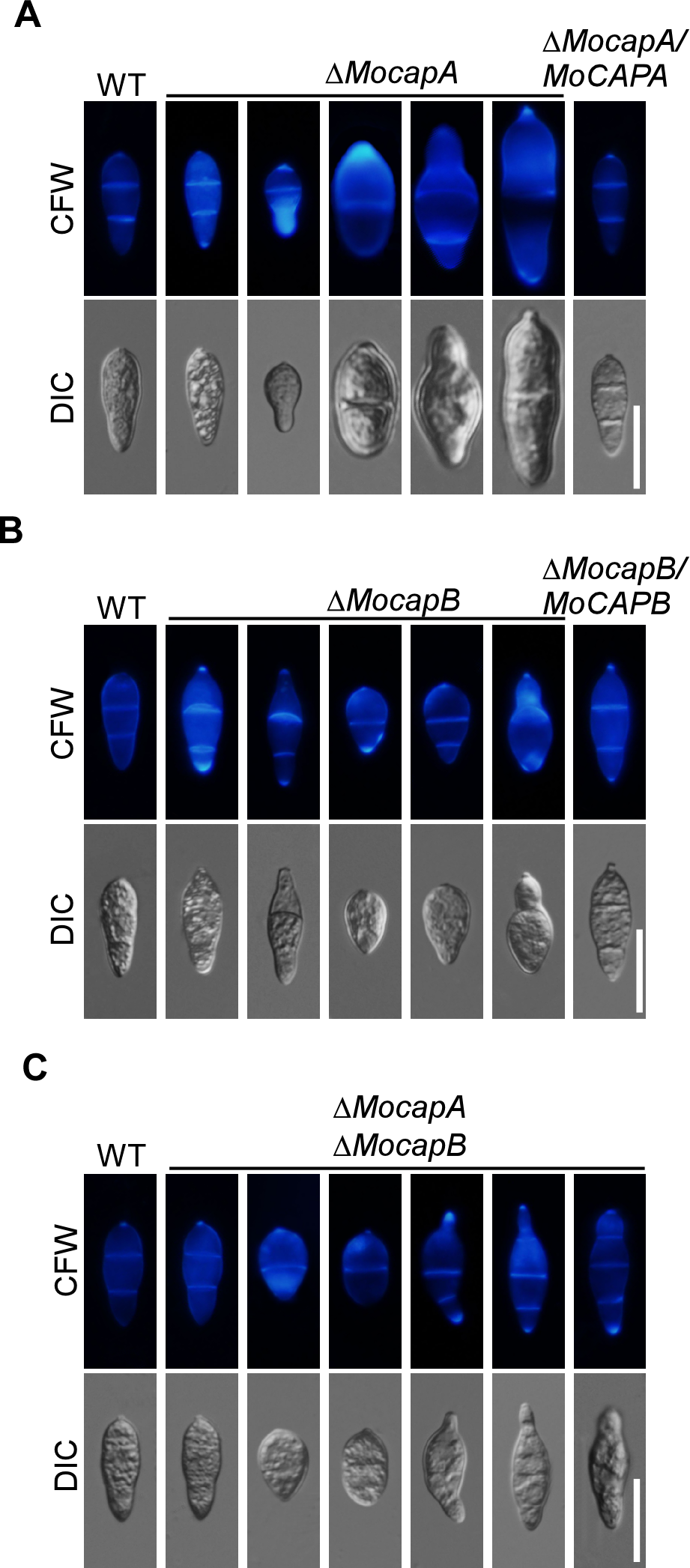

Supplement: S6 Fig — (A-C) Conidia shape comparison. Conidia of the indicated strains were harvested from SDC medium, stained with calcofluor white, and observed by fluorescence microscope. Bar = 10 μm. (TIF) [file pgen.1006814.s006.tif]

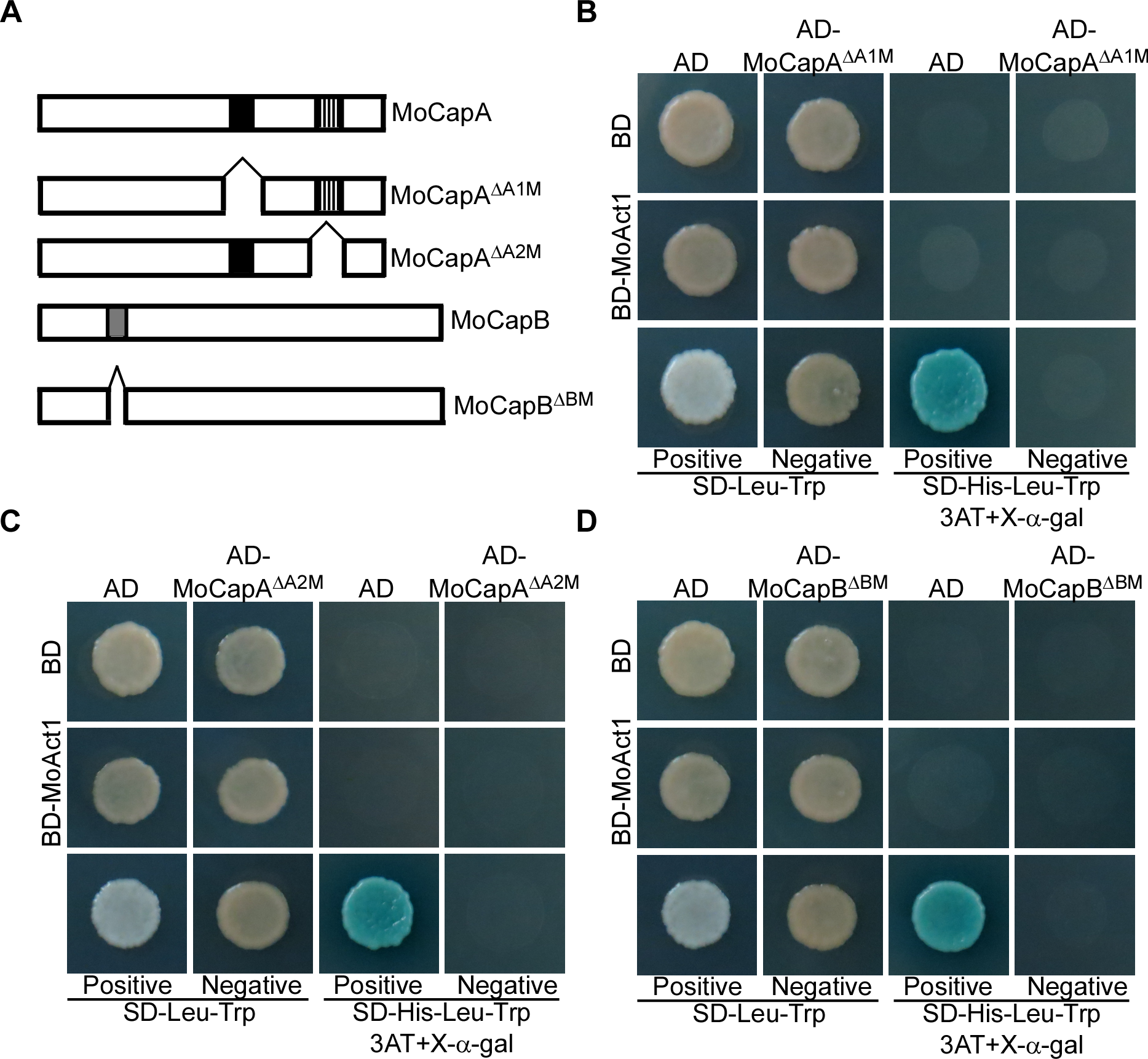

Supplement: S7 Fig — (A) Schematic representation of motifs deleted in MoCapA and MoCapB. (B, C and D) MoCapAΔA1M, MoCapAΔA2M and MoCapBΔBM do not interacts with MoAct1. Yeast transformants expressing bait (pGBKT7) and prey (pGADT7) constructs were assayed for growth on SD-His-Leu-Trp plates added with 1mM 3AT and β-galactosidase (LacZ) activities with positive and negative control. MoCapAΔA1M (MoCapAΔVHYYEDGNV, MoCapA without F-actin-capping A1 motif), MoCapAΔA2M (MoCapAΔKGLRRQLPVTR, MoCapA without F-actin-capping A2 motif), MoCapBΔBM (MoCapBΔCDYNRD, MoCapB without F-actin-capping B motif). (TIF) [file pgen.1006814.s007.tif]

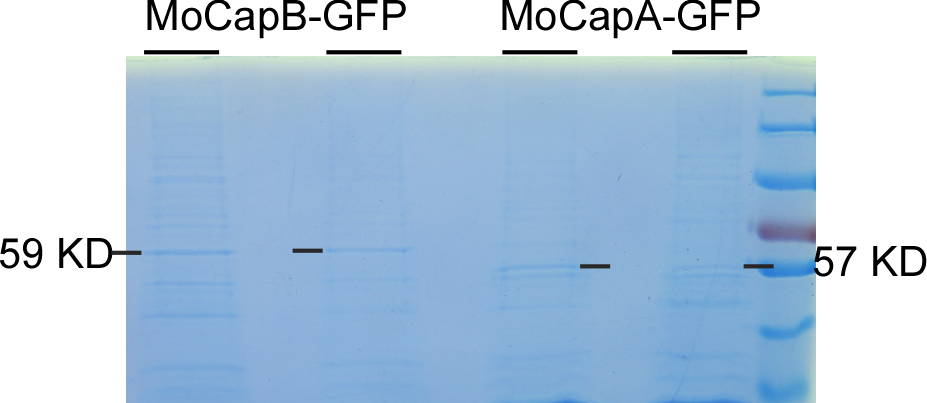

Supplement: S8 Fig — MoCapA-GFP and MoCapB-GFP protein were purified form MoCAPA-GFP and MoCAPB-GFP expressing cells. MoCapA-GFP and MoCapB-GFP protein were separated by SDS-PAGE and subject for LC-MS/MS analysis as mentioned in the materials and methods. (TIF) [file pgen.1006814.s008.tif]

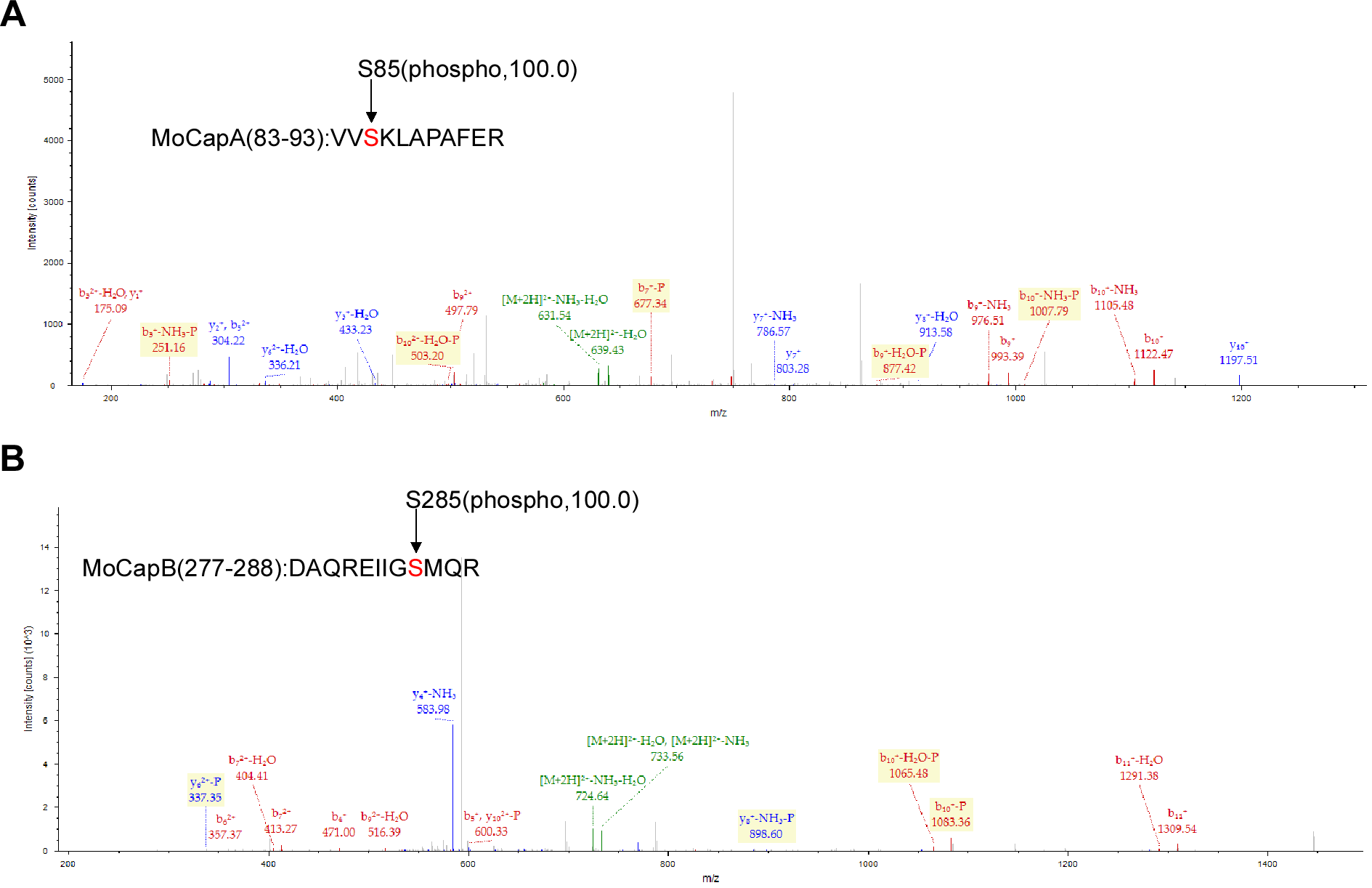

Supplement: S9 Fig — (A and B) Mass spectrometry showed that the serine at the 85th residue (S85) of MoCapA and the serine at the 285th residue (S285) of MoCapB were phosphorylated. An analysis of a phosphopeptide from purified MoCapA-GFP and MoCapB-GFP expressed in the wild type. The product ion spectrum of m/z 648.8 identifies the peptide as mono-phosphorylated VVSpKLAPAFER and pinpoints serine 85 as the site of phosphorylation of MoCapA. For MoCapA, [M+2H] represented the identified peptide fragment with Pi group (corresponding molecular weight 1296.36). When one amino acid (the 1st one is Arg) was removed from the carboxyl terminal of the peptide fragment during MS analysis, b10+ (with 1 positive charge) was produced with a molecular weight of 1122.47. Then, amino acid residues or NH3/H2O/Pi groups were subsequently removed to produce b9+, b10+-NH3, b10+-NH3-Pi, and so on. When amino acid residues or NH3/H2O/Pi were removed from the amino terminal, y series fragments were produced. From the molecular weight y8+-H2O, we can calculated that a phosphorylated serine was presented in the fragment (MW of y8 is equal to y10 with Val and Ser-Pi residues removed). Also, from the molecular weight of b3+-NH3-P and b32+-H2O, we can calculated that a phosphorylated serine was presented. The product ion spectrum of m/z 742.3 identifies the peptide as mono-phosphorylated DAQREIIGSpMQR and pinpoints serine 285 as the site of phosphorylation of MoCapB. By the same calculation process, the phosphorylated serine in MoCapB (MW of b7 is equal to b8 with Gly and Ser-Pi residues removed) was calculated. (TIF) [file pgen.1006814.s009.tif]
