# Supplementary material for: MoCAP proteins regulated by MoArk1-mediated phosphorylation coordinate endocytosis and actin dynamics to govern development and virulence of Magnaporthe oryzae
Source: PLoS Genet. 2017 May 25;13(5):e1006814. doi: 10.1371/journal.pgen.1006814 (PMC5466339; doi:10.1371/journal.pgen.1006814)
Supplement: S1 Table — (DOCX) [file pgen.1006814.s013.docx]

| **S1 Table. Putative MoArk1-interacting proteins identified by affinity purification** | | | |
| --- | --- | --- | --- |
| Gene ID | Predicted function | No. of unique peptides | |
|  |  | Rep1 | Rep2 |
| MGG_05193.6 | cell division cycle protein 48 | 6 | 3 |
| MGG_07768.6 | clathrin heavy chain | 4 | 2 |
| MGG_03838.6 | ser/thr protein phosphatase family protein | 3 | 1 |
| MGG_06358.6 | amylase-binding protein AbpA | 3 | 2 |
| MGG_03087.6 | cell division control protein 11 | 3 | 2 |
| MGG_04745.6 | Arp2/3 complex 34 kDa subunit | 1 | 2 |
| MGG_01521.6 | cell division control protein 3 | 2 | 1 |
| MGG_06649.6 | EF hand domain-containing protein | 2 | 1 |
| MGG_06180.6 | endocytosis and cytoskeletal organization protein | 1 | 2 |
| MGG_09902.6 | F-actin-capping protein subunit beta | 2 | 2 |
| MGG_08547.6 | serine/threonine-protein kinase Srk1 | 1 | 1 |
| MGG_06367.6 | vesicular integral-membrane protein Vip36 | 1 | 1 |
| MGG_06361.6 | dynamin-A | 2 | 1 |
| MGG_12818.7 | F-actin-capping protein subunit alpha | 2 | 2 |
| MGG_06241.6 | vacuolar protein sorting-associated protein 21 | 2 | 1 |
| MGG_07859 | hypothetical protein | 1 | 2 |
| MGG_01569.6 | conserved hypothetical protein | 2 | 1 |
| MGG_08098.6 | conserved hypothetical protein | 2 | 2 |
| MGG_10856 | hypothetical protein | 1 | 2 |
| MGG_06958.6 | hsp70-like protein | 7 | 3 |
| MGG_11513.6 | heat shock protein Ssb1 | 3 | 4 |
| MGG_06860.6 | coatomer subunit beta | 1 | 5 |
| MGG_14971.6 | elongation factor 3 | 3 | 7 |
| MGG_04719.6 | WD repeat-containing protein 38 | 2 | 2 |
| MGG_12839.6 | conserved hypothetical protein | 2 | 2 |
| MGG_05626.6 | cytoskeleton assembly control protein Sla1p | 1 | 1 |
| MGG_01722.6 | adenylyl cyclase-associated protein | 1 | 2 |
