# Supplementary material for: MoCAP proteins regulated by MoArk1-mediated phosphorylation coordinate endocytosis and actin dynamics to govern development and virulence of Magnaporthe oryzae
Source: PLoS Genet. 2017 May 25;13(5):e1006814. doi: 10.1371/journal.pgen.1006814 (PMC5466339; doi:10.1371/journal.pgen.1006814)
Supplement: S2 Table — (DOCX) [file pgen.1006814.s014.docx]

| **S2 Table. Statistical analysas of the growth and conidiation of the phosphorylation site mutants** | | |
| --- | --- | --- |
| Gene ID | Growth (cm) ^α^ | Conidiation (×10^4^/cm^2^) ^β^ |
| WT | 4.8±0.1A | 21.9±1.0A |
| Δ*MocapA* | 2.0±0.1D | 5.1±0.6D |
| Δ*MocapA/MoCAPA*^S85A^ | 4.1±0.1B | 15.8±0.2B |
| Δ*MocapA/MoCAPA*^S85D^ | 2.6±0.1C | 12.1±1.4C |
| Δ*MocapB* | 1.9±0.1D | 5.4±1.0D |
| Δ*MocapB/MoCAPB*^S285A^ | 4.2±0.2B | 14.6±0.7B |
| Δ*MocapB/MoCAPB*^S285D^ | 2.9±0.1C | 11.4±0.8C |

α. Diameter of hyphal radii at day 7 after incubation on CM agar plates at room temperature.

β. Number of conidia harvested from a 9 cm SDC plate at day 10 after incubation at room temperature.

The different capital letters in a column show signiﬁcant difference (*P*<0.01)
