# Supplementary material for: MoCAP proteins regulated by MoArk1-mediated phosphorylation coordinate endocytosis and actin dynamics to govern development and virulence of Magnaporthe oryzae
Source: PLoS Genet. 2017 May 25;13(5):e1006814. doi: 10.1371/journal.pgen.1006814 (PMC5466339; doi:10.1371/journal.pgen.1006814)
Supplement: S3 Table — (DOCX) [file pgen.1006814.s015.docx]

| **S3 Table. Comparison of the growth and conidiation among Δ*Moark1* and phosphorylation site mutants** | | |
| --- | --- | --- |
| Gene ID | Growth (cm) ^α^ | Conidiation (×10^4^/cm^2^) ^β^ |
| WT | 4.8±0.1A | 21.9±1.0A |
| Δ*Moark1* | 3.6±0.1C | 5.4±0.5C |
| Δ*Moark1/MoCAPA*^S85A^ | 4.1±0.2B | 11.6±0.3B |
| Δ*Moark1/MoCAPA*^S85D^ | 3.2±0.2D | 5.1±0.4C |
| Δ*Moark1/MoCAPB*^S285A^ | 4.1±0.3B | 13.0±0.9B |
| Δ *Moark1/MoCAPB*^S285D^ | 2.9±0.1D | 4.2±0.8C |

α. Diameter of hyphal radii at day 7 after incubation on CM agar plates at room temperature.

β. Number of conidia harvested from a 9 cm SDC plate at day 10 after incubation at room temperature.

The different capital letters in a column show signiﬁcant difference (*P*<0.01)
