# Supplementary material for: MoCAP proteins regulated by MoArk1-mediated phosphorylation coordinate endocytosis and actin dynamics to govern development and virulence of Magnaporthe oryzae
Source: PLoS Genet. 2017 May 25;13(5):e1006814. doi: 10.1371/journal.pgen.1006814 (PMC5466339; doi:10.1371/journal.pgen.1006814)
Supplement: S4 Table — (DOC) [file pgen.1006814.s016.doc]

**S4 Table.** Primers used in this study

| **Primer name** | **Sequence (5’-3’)** | **Remark** |
| --- | --- | --- |
| **MoCapA** | | |
| MGG_12818YTHQCF | CGGAATTCATGACCAAGTCTACCGAGGGTAGGTG | Construction of pGADT7*-MoCAPA* construct |
| MGG_12818YTHQCR | CGGGATCCCTAGCCCTGACGCCTGCCC | Construction of pGADT7*-MoCAPA* construct |
| MGG_12818pulldownF | CGGGATCCATGACCAAGTCTACCGAGGGTAGGTG | Construction of pET-32a-*MoCAPA* construct |
| MGG_12818pulldownR | CGGAATTCCTAGCCCTGACGCCTGCCC | Construction of pET-32a-*MoCAPA* construct |
| MGG_12818phz65F | CGACTCACTATAGGGCGAATTGGGTACTCAAATTG CACGCCGCTGTGTGAGCAAGGAATTATG | Construction of *MoCAPA-*YFP^N^ |
| MGG_12818phz65R | GCTCACCATCGTGGCGATGGAGCG GCCCTGACGCCTGCCCCCGCCGATAT | Construction of *MoCAPA-*YFP^N^ |
| MGG_12818RTF | CCAGGAGGAAGCCAGAAGGTCA | quantitative RT-PCR analysis *MoCAPA* |
| MGG_12818RTR | TCGCCACCGTCCGTTCACA | quantitative RT-PCR analysis *MoCAPA* |
| MGG_12818comF | ACTCACTATAGGGCGAATTGGGTACTCAAATTGGTTCACGCCGCTGTGTGAGCAAGGAATTATG | *MoCAPA* complementation |
| MGG_12818comR | CACCACCCCGGTGAACAGCTCCTCGCCCTTGCTCACGCCCTGACGCCTGCCCCCGCCGATAT | *MoCAPA* complementation |
| MGG_12818A1F | ACTCACTATAGGGCGAATTGGGTACTCAAATTGGTTCACGCCGCTGTGTGAGCAAGGAATTATG | Construction of *MoCAPA*^ΔVHYYEDGNV^-GFP construct |
| MGG_12818A1R | GTCGACTTTGATCGAACCGTCGATG | Construction of *MoCAPA*^ΔVHYYEDGNV^-GFP construct |
| MGG_12818B1F | GGAGCCATCGACGGTTCGATCAAAGTCGACCGACTTCTGACGGACAAGGCCACAA | Construction of *MoCAPA*^ΔVHYYEDGNV^-GFP construct |
| MGG_12818B1R | CACCACCCCGGTGAACAGCTCCTCGCCCTTGCTCACGCCCTGACGCCTGCCCCCGCCGATAT | Construction of *MoCAPA*^ΔVHYYEDGNV^-GFP construct |
| MGG_12818A2F | ACTCACTATAGGGCGAATTGGGTACTCAAATTGGTTCACGCCGCTGTGTGAGCAAGGAATTATG | Construction of *MoCAPA*^ΔKGLRRQLPVTR^-GFP construct |
| MGG_12818A2R | GAAGGCCCCTTCGCTGAGGTCTGTA | Construction of *MoCAPA*^ΔKGLRRQLPVTR^-GFP construct |
| MGG_12818B2F | GGCTTTACAGACCTCAGCGAAGGGGCCTTCCAGAAGATTGAGTGGGACAAGGTCG | Construction of *MoCAPA*^ΔKGLRRQLPVTR^-GFP construct |
| MGG_12818B2R | CACCACCCCGGTGAACAGCTCCTCGCCCTTGCTCACGCCCTGACGCCTGCCCCCGCCGATAT | Construction of *MoCAPA*^ΔKGLRRQLPVTR^-GFP construct |
| MGG_12818-S85AF1 | ACTCACTATAGGGCGAATTGGGTACTCAAATTGGTTCACGCCGCTGTGTGAGCAAGGAAT | Construction of *MoCAPA*^S85A^-GFP construct |
| MGG_12818-S85AR1 | AACAACCTTCGCATCGCCTGACGAT | Construction of *MoCAPA*^S85A^-GFP construct |
| MGG_12818-S85AF2 | TCGATATCGTCAGGCGATGCGAAGGTTGTTGCAAAGCTCGCGCCCGCATTCGAGA | Construction of *MoCAPA*^S85A^-GFP construct |
| MGG_12818-S85AR2 | CACCACCCCGGTGAACAGCTCCTCGCCCTTGCTCACGCCCTGACGCCTGCCCCCGCCGATAT | Construction of *MoCAPA*^S85A^-GFP construct |
| MGG_12818-S85DF1 | ACTCACTATAGGGCGAATTGGGTACTCAAATTGGTTCACGCCGCTGTGTGAGCAAGGAAT | Construction of *MoCAPA*^S85D^-GFP construct |
| MGG_12818-S85DR1 | AACAACCTTCGCATCGCCTGACGAT | Construction of *MoCAPA*^S85D^-GFP construct |
| MGG_12818-S85DF2 | TCGATATCGTCAGGCGATGCGAAGGTTGTTGACAAGCTCGCGCCCGCATTCGAGA | Construction of *MoCAPA*^S85D^-GFP construct |
| MGG_12818-S85DR2 | CACCACCCCGGTGAACAGCTCCTCGCCCTTGCTCACGCCCTGACGCCTGCCCCCGCCGATAT | Construction of *MoCAPA*^S85D^-GFP construct |
| **MoCapB** | | |
| MGG_09902YTHQCF | CGGAATTCATGGCTGCAGACCCTTTCGATTC | Construction of pGADT7*-MoCAPB* construct |
| MGG_09902YTHQCR | CGGGATCCTCACCTCTGCATGCTCCCTATGATC | Construction of pGADT7*-MoCAPB* construct |
| MGG_09902pulldownF | CGGGATCCATGGCTGCAGACCCTTTCGATTC | Construction of pET-32a-*MoCAPB* construct |
| MGG_09902pulldownR | CGGAATTCTCACCTCTGCATGCTCCCTATGATC | Construction of pET-32a-*MoCAPB* construct |
| MGG_09902phz65F | CGACTCACTATAGGGCGAATTGGGTACTCAAATTGCTAAGGAGAACTGCGGGAACTGG | Construction of *MoCAPB-*YFP^N^ |
| MGG_09902phz65R | GCTCACCATCGTGGCGATGGAGCGCCTCTGCATGCTCCCTATGATCT | Construction of *MoCAPB-*YFP^N^ |
| MGG_09902RTF | GGCTTTGCTGGCGTGGTGCT | quantitative RT-PCR analysis *MoCAPB* |
| MGG_09902RTR | ACGGTCGATGTCAGTCGGTAAGTTGT | quantitative RT-PCR analysis *MoCAPB* |
| MGG_09902comF | ACTCACTATAGGGCGAATTGGGTACTCAAATTGGTTCTAAGGAGAACTGCGGGAACTGG | *MoCAPB* complementation |
| MGG_09902comR | CACCACCCCGGTGAACAGCTCCTCGCCCTTGCTCACCCTCTGCATGCTCCCTATGATCT | *MoCAPB* complementation |
| MGG_09902AF | ACTCACTATAGGGCGAATTGGGTACTCAAATTGGTTCTAAGGAGAACTGCGGGAACTGG | Construction of *MoCAPB*^ΔCDYNRD^-GFP construct |
| MGG_09902AR | CAGCAGGTATTCGCGGCCCGTT | Construction of *MoCAPB*^ΔCDYNRD^-GFP construct |
| MGG_09902BF | TGCAAGCAAACGGGCCGCGAATACCTGCTGGGCGACTCCTACCGCTCGCCTTGGT | Construction of *MoCAPB*^ΔCDYNRD^-GFP construct |
| MGG_09902BR | CACCACCCCGGTGAACAGCTCCTCGCCCTTGCTCACCCTCTGCATGCTCCCTATGATCT | Construction of *MoCAPB*^ΔCDYNRD^-GFP construct |
| MGG_09902S285A-F | ACTCACTATAGGGCGAATTGGGTACTCAAATTGGTTCTAAGGAGAACTGCGGGAACTGGCT | Construction of *MoCAPB*^S285A^-GFP construct |
| MGG_09902S285A-R | CACCACCCCGGTGAACAGCTCCTCGCCCTTGCTCACCCTCTGCATGGCCCCTATGATCTC | Construction of *MoCAPB*^S285A^-GFP construct |
| MGG_09902S285D-F | ACTCACTATAGGGCGAATTGGGTACTCAAATTGGTTCTAAGGAGAACTGCGGGAACTGGCT | Construction of *MoCAPB*^S285D^-GFP construct |
| MGG_09902S285D-R | CACCACCCCGGTGAACAGCTCCTCGCCCTTGCTCACCCTCTGCATGTCCCCTATGATCTC | Construction of *MoCAPB*^S285D^-GFP construct |
| **MoArk1 and others** |  |  |
| MoArk1flagF | CTATAGGGCGAATTGGGTACTCAAATTGGTTACCTGGCTGCCTTCATCGTCATCG | Construction of *MoARK1*-3xFLAG |
| MoArk1flagR | CTTTATAATCACCGTCATGGTCTTTGTAGTCACGTTCCCGGTCCCTCCCGC | Construction of *MoARK1*-3xFLAG |
| MoArk1YTHQCF | CGGAATTCATGTTGGCGTCGGCCGCTAGG | Construction of pGBKT7*-MoARK1* |
| MoArk1YTHQCR | CGGGATCCTCAACGTTCCCGGTCCCTCCC | Construction of pGBKT7*-MoARK1* |
| MoArk1pulldownF | CGGGATCCATGTTGGCGTCGGCCGCTAGG | Construction of pGEX4T-2-*MoARK1* |
| MoArk1pulldownR | CGGAATTCTCAACGTTCCCGGTCCCTCCC | Construction of pGEX4T-2-*MoARK1* |
| MoArk1phz68F | CGACTCACTATAGGGCGAATTGGGTACTCAAATTG CCTCATGTCTGGAGCCCGCATG | Construction of *MoARK1-*YFP^C^ |
| MoArk1phz68R | GTTCGGGATCTTGCAGGCCGGGCG ACGTTCCCGGTCCCTCCCGC | Construction of *MoARK1-*YFP^C^ |
| RFPh1F | AAGACTGAAAAGGCCGAGGCACCCGCCGCAATGGCCTCCTCCGAGGACGTCATCA | Construction of pYF11*-H1-*RFP |
| RFPh1R | TTACTTGTACAGCTCGTCCATGCCGAGAGTTTAGGCGCCGGTGGAGTGGCG | Construction of pYF11*-H1-*RFP |
| H1RfpF | TTTCGTAGGAACCCAATCTTCAAAATGCCTCCCAAGAAGGAAACCAAGG | Construction of pYF11*-H1-*RFP |
| H1RfpR | TGCGGCGGGTGCCTCGGCCTTTTC | Construction of pYF11*-H1-*RFP |
